# Supplementary material for: A tough nitric oxide-eluting hydrogel coating suppresses neointimal hyperplasia on vascular stent
Source: Nat Commun. 2021 Dec 6;12:7079. doi: 10.1038/s41467-021-27368-4 (PMC8648853; doi:10.1038/s41467-021-27368-4)
Supplement: Supplementary file 1 — Supplementary Information [file 41467_2021_27368_MOESM1_ESM.pdf]

A tough nitric oxide-eluting hydrogel coating suppresses neointimal hyperplasia on vascular stent

Yin Chen[1,2], Peng Gao[3], Lu Huang[1], Xing Tan[3], Ningling Zhou[3], Tong Yang[3], Hua Qiu[3], Xin Dai[2], Sean Michael[2], Qiufen Tu[3], Nan Huang[3], Zhihong Guo[2], Jianhua Zhou[1,4]*, Zhilu Yang[3]* and Hongkai Wu[2]*

[1]School of Biomedical Engineering, Sun Yat-sen University, Shenzhen 518107, China.
[2]Department of Chemistry, The Hong Kong University of Science and Technology, Hong Kong, China.
[3]Key Laboratory of Advanced Technologies of Materials, Ministry of Education, School of Materials Science and Engineering, Southwest Jiaotong University, Chengdu 610031, China.
[4]Division of Engineering in Medicine, Brigham and Women's Hospital, Harvard Medical School, Cambridge, MA 02139, USA.
These authors contributed equally: Yin Chen, Peng Gao
*e-mail: zhoujh33@mail.sysu.edu.cn; zhiluyang1029@swjtu.edu.cn; chhkwu@ust.hk

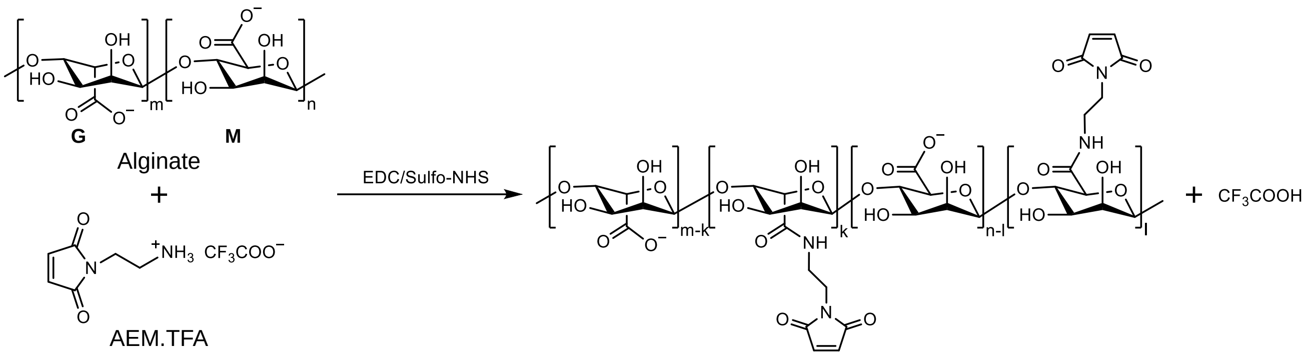

Supplementary Fig. 1 | Schematic for the synthesis of maleimidyl alginate (A-M). A-M was synthesized by the coupling reaction between pristine alginate and N-(2-aminoethyl)maleimide trifluoroacetate (AEM.TFA) via aqueous carbodiimide chemistry.

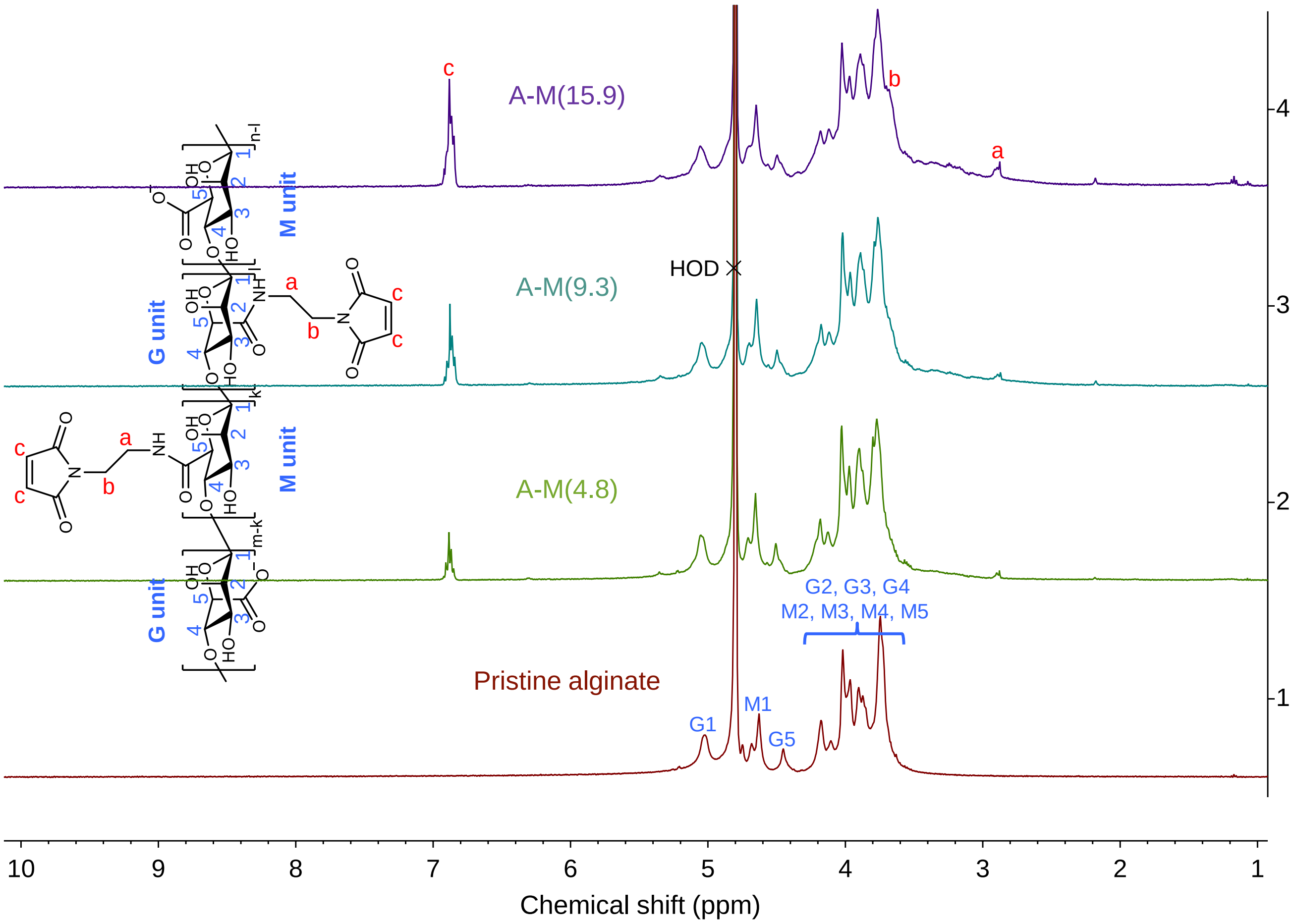

Supplementary Fig. 2 | ¹H-NMR spectra of pristine alginate and maleimidyl alginate(A-M) of varying degrees of modification (DM). The chemical structure of A-M is presented with the assignment of peaks to the corresponding protons.

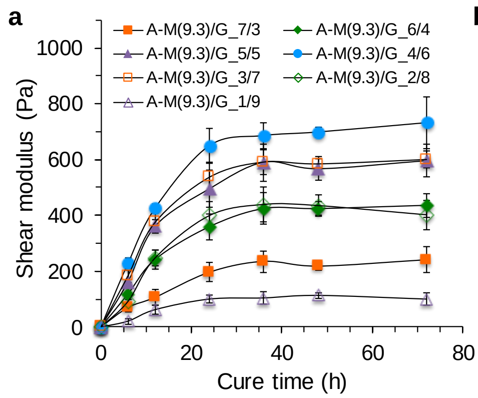

	7/3	6/4	5/5	4/6	3/7	2/8	1/9
7/3	1	0.0076	0.000019	3.8e-007	0.000017	0.031	0.071
6/4	0.0076	1	0.032	0.00015	0.027	0.98	0.000036
5/5	0.000019	0.032	1	0.086	1.0	0.0079	3.3e-007
4/6	3.8e-007	0.00015	0.086	1	0.10	0.000044	1.4e-008
3/7	0.000017	0.027	1.0	0.10	1	0.0066	3e-007
2/8	0.031	0.98	0.0079	0.000044	0.0066	1	0.00012
1/9	0.071	0.000036	3.3e-007	1.4e-008	3e-007	0.00012	1

Supplementary Fig. 3 | Cure kinetics of maleimidyl alginate/gelatin (A-M/G) hybrid hydrogels at 37 °C characterized by dynamic mechanical test. a, Cure kinetics of the hydrogels formulated at varying mass ratios of A-M(9.3) to gelatin (mean ± SD, n = 3 independent samples). **b**, Comparison in shear modulus among them after 72 h (mean ± SD, n = 3 independent samples). The matrix shows the significance of difference among various groups determined by one-way analysis of variance (ANOVA) with Tukey post-hoc test. The mass concentration of the precursor solution (pH~7.5) was 10 w/v% for both biopolymers. The dynamic mechanical test was performed at 37 °C, 5 % strain and 1 rad s^{-1} angular frequency.

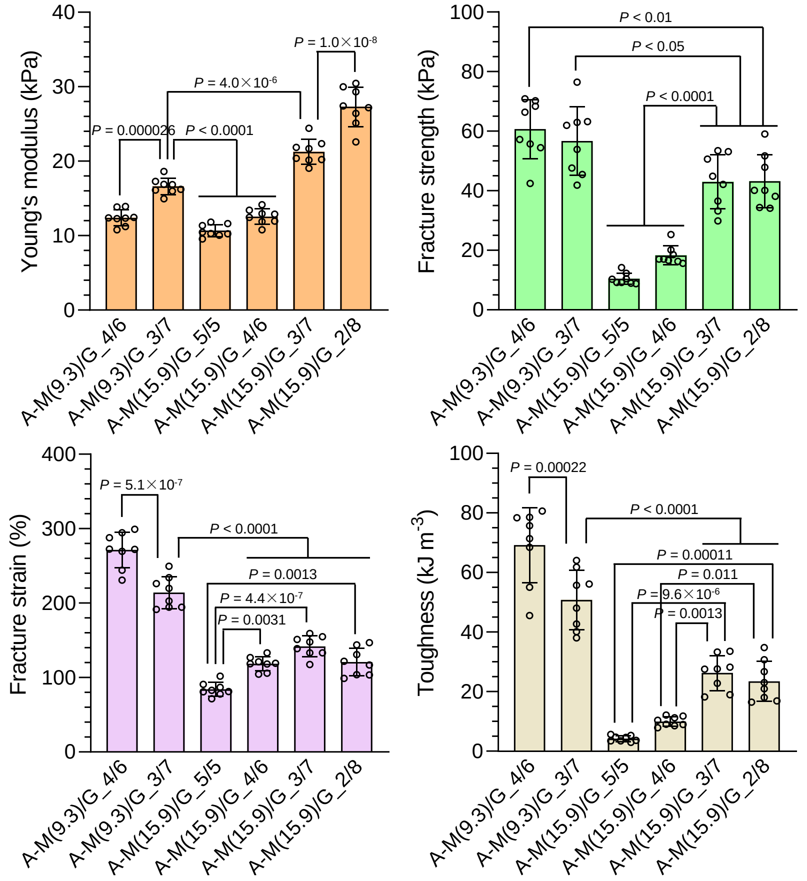

Supplementary Fig. 4 | Summary on the tensile properties of maleimidyl alginate/gelatin (A-M/G) hydrogels at ambient temperature. (mean ± SD, n = 8 independent samples) The mass concentration of the precursor solution (pH~7.5) was 15 w/v% for both biopolymers. One-way ANOVA with Tukey post-hoc test was performed to determine the difference among them.

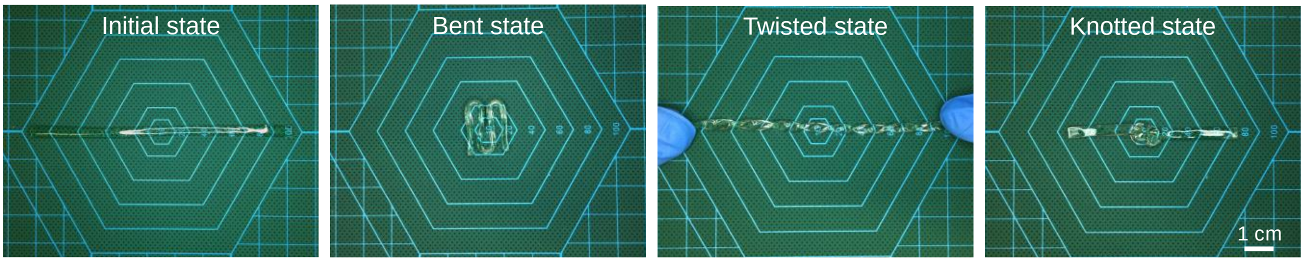

Supplementary Fig. 5 | Manipulation of A-M(9.3)/G_4/6 hydrogel in various manners.

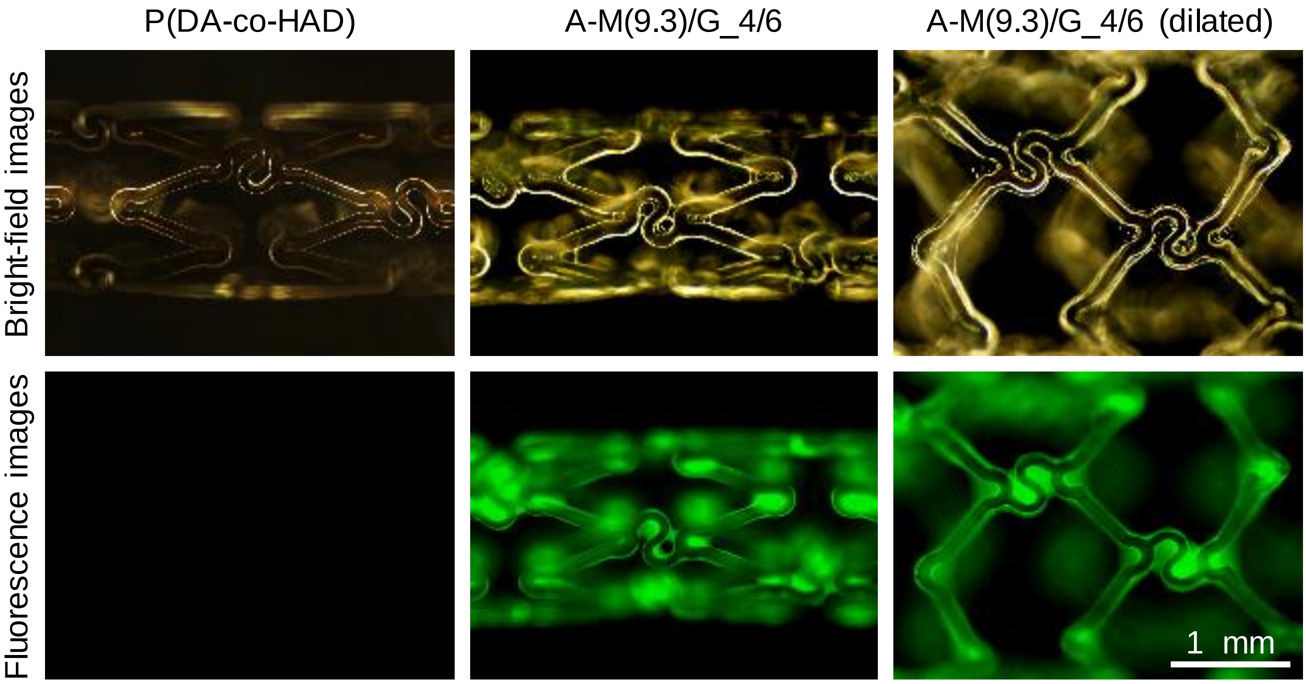

Supplementary Fig. 6 | Bright-field images and fluorescence images of vascular stents coated with poly(dopamine-co-hexanediamine) (P(DA-co-HDA)) or A-M(9.3)/G_4/6 hydrogel.

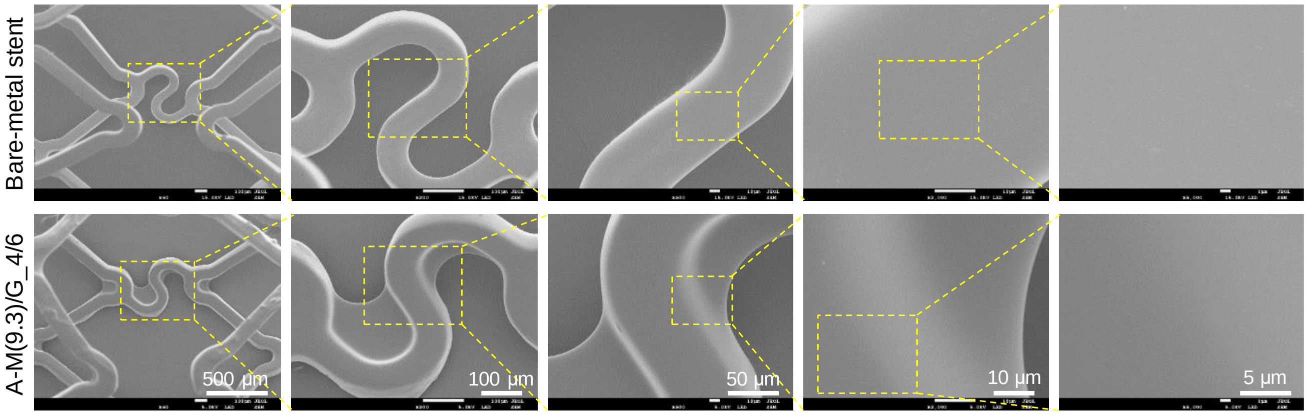

Supplementary Fig. 7 | Scanning electron microscopy (SEM) images showing the strut surfaces of a bare-metal (316L stainless steel) stent and a stent coated with A-M(9.3)/G_4/6 hydrogel after balloon dilation at 37 °C. Two independent pairs of samples were tested with similar results.

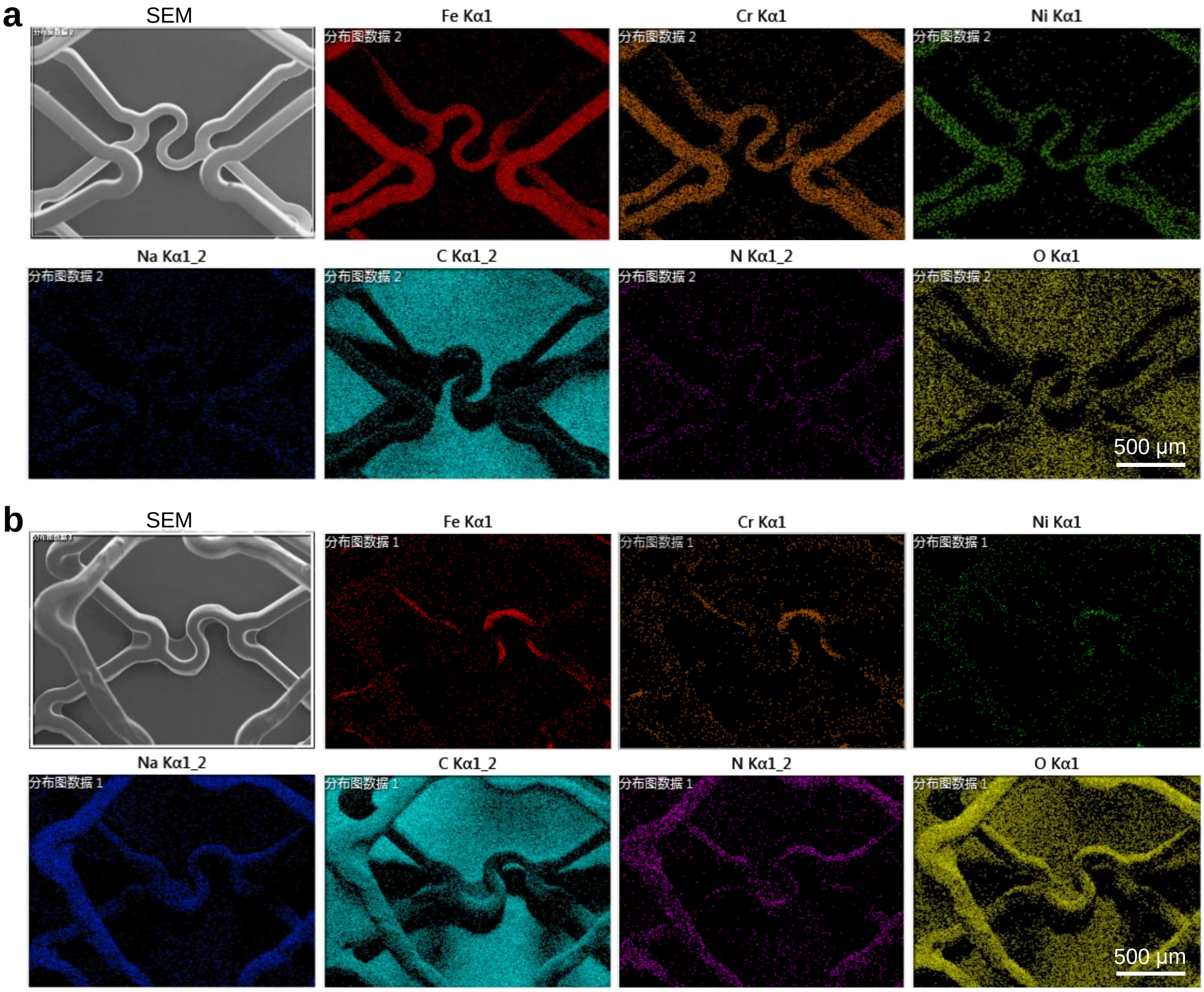

Supplementary Fig. 8 | Energy-dispersive X-ray spectroscopy (EDS) mapping on the strut surfaces of a bare-metal stent (316L stainless steel) (a) and a stent coated with A-M(9.3)/G_4/6 hydrogel (b) after balloon dilation at 37 °C.

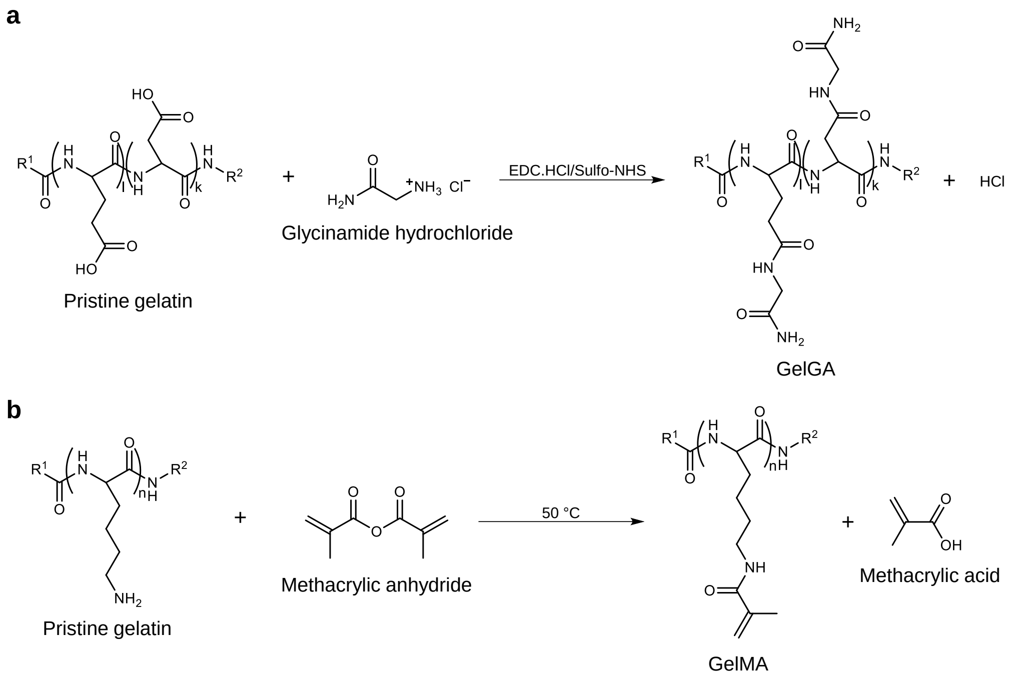

Supplementary Fig. 9 | Schematic for the syntheses of gelatin glycinamidate (GelGA) and gelatin methacrylate (GelMA). a, Synthesis of GelGA through the conjugation of glycinamide to the glutamic acid and aspartic acid residues of gelatin. **b,** Synthesis of GelMA via the coupling reaction between the lysine residue of gelatin and methacrylic anhydride.

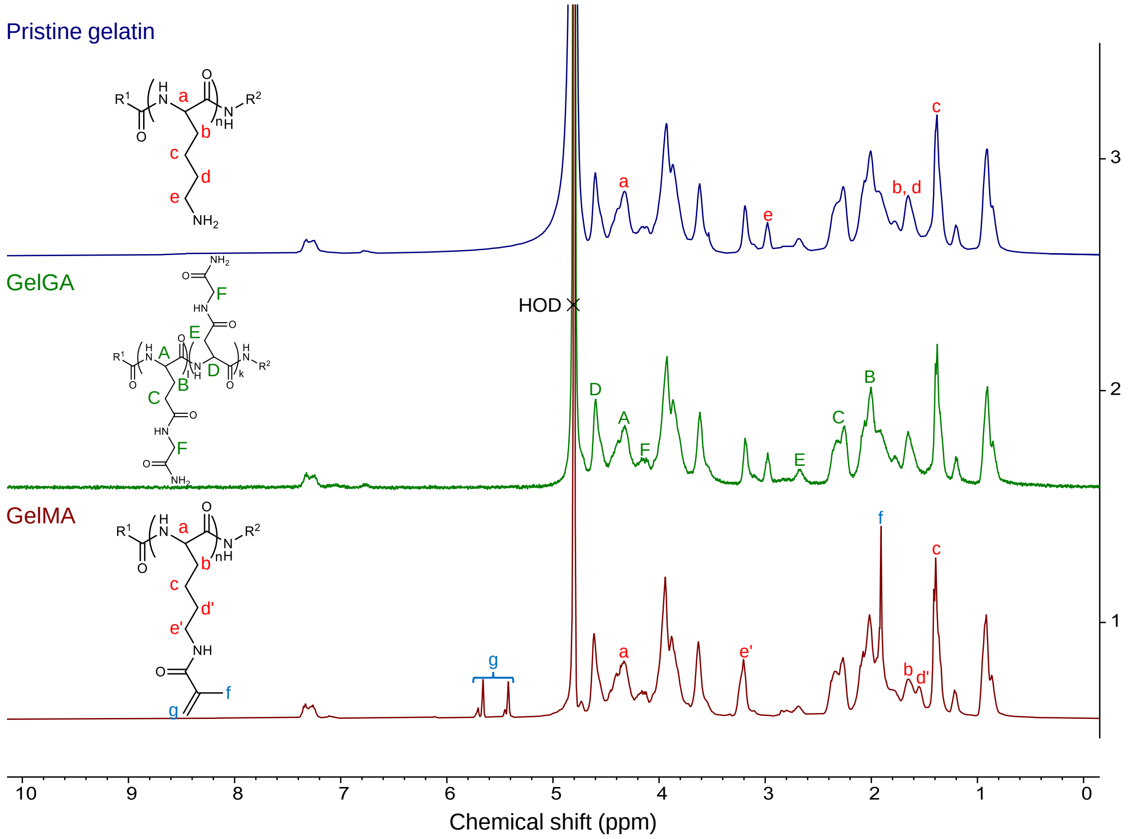

Supplementary Fig. 10 | ^{1}H-NMR spectra of pristine gelatin, gelatin glycinamidate (GelGA) and gelatin methacrylate (GelMA). The chemical structures of the modified amino acid residues are presented with the assignment of peaks to the corresponding protons.

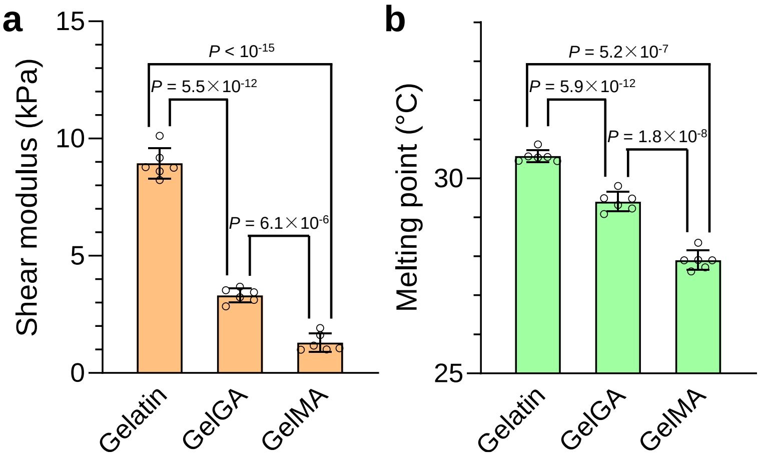

Supplementary Fig. 11 | Effects of chemical modifications on the gelation potency of gelatin. a, comparison in shear modulus among pristine gelatin, gelatin glycinamidate (GelGA) and gelatin methacrylate (GelMA) hydrogels at ambient temperature. (mean ± SD, n = 6 independent samples) **b**, Comparison in melting points among the hydrogels. (mean ± SD, n = 6 independent samples) The melting point is defined as the temperature at which the shear modulus of a hydrogel is reduced by a half from that at ambient temperature. The mass concentration of the precursor solution (pH~7.5) was 15 w/v% for all biopolymers. One-way ANOVA with Tukey post-hoc test was performed to determine the difference among various hydrogels.

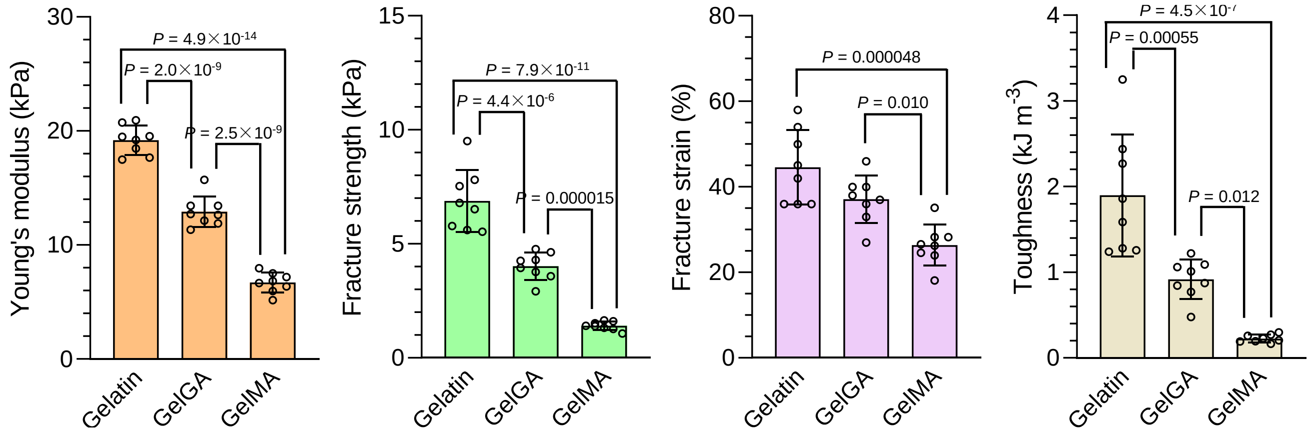

Supplementary Fig. 12 | Comparison in tensile property among pristine gelatin, gelatin glycinamidate (GelGA) and gelatin methacrylate (GelMA) hydrogels at ambient temperature. (mean ± SD, n = 8 independent samples) One-way ANOVA with Tukey post-hoc test was performed to determine the difference among various hydrogels.

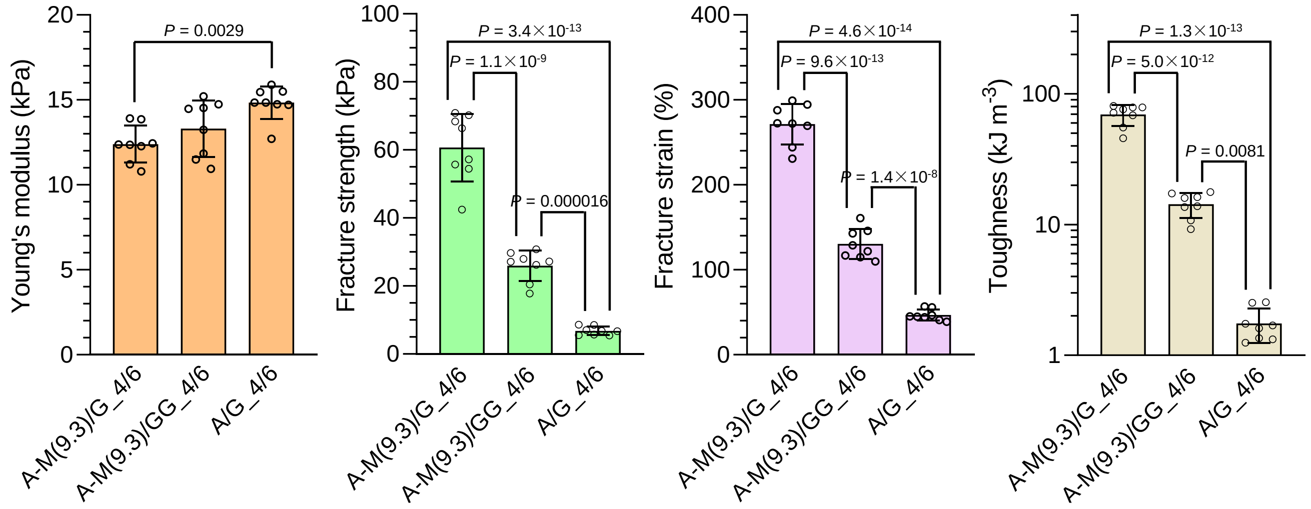

Supplementary Fig. 13 | Comparison in tensile property among A-M(9.3)/G_4/6, A-M(9.3)/GG_4/6 and A/G_4/6 hydrogels. (mean ± SD, *n* = 8 independent samples) One-way ANOVA with Tukey post-hoc test was performed to determine the difference among various hydrogels.

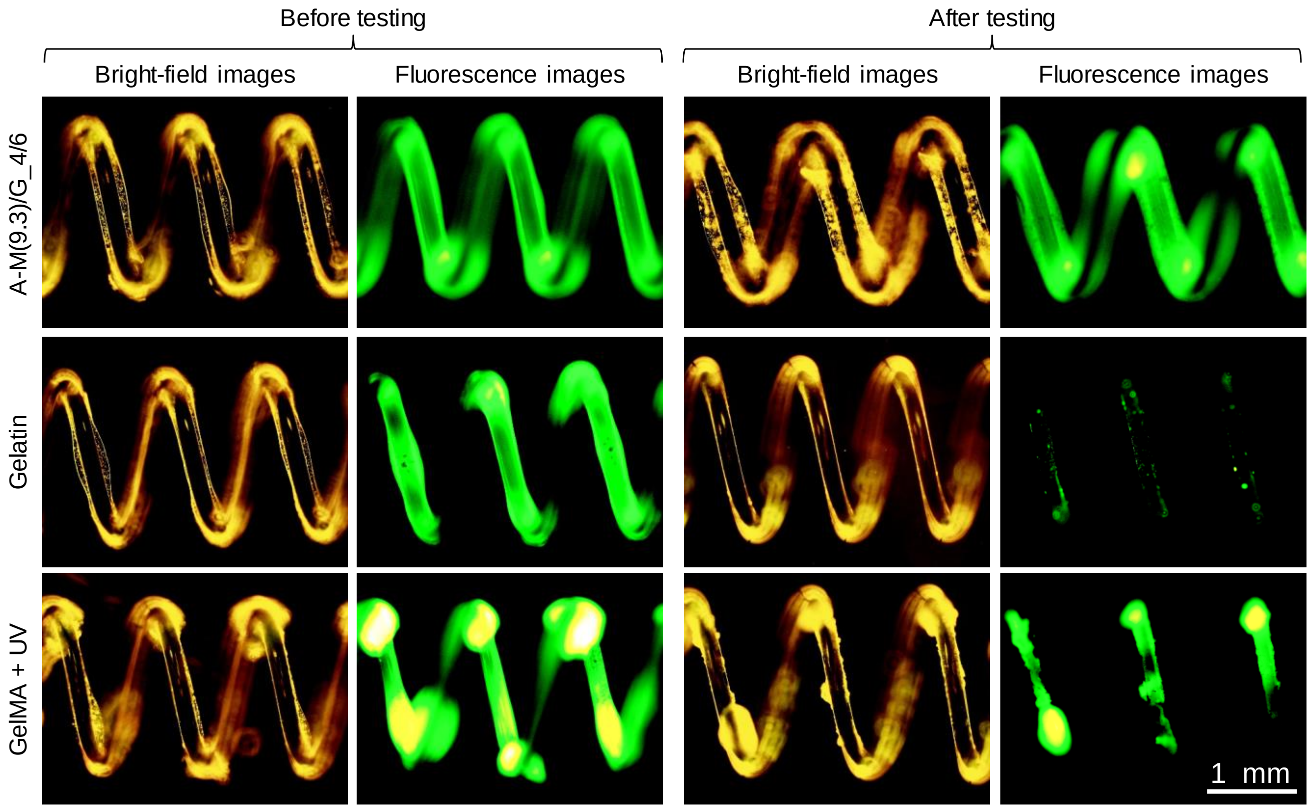

Supplementary Fig. 14 | Optical images of metal springs coated with A-M(9.3)/G_4/6, gelatin or gelatin methacrylate (GelMA, illuminated by UV for 30 min) hydrogel before and after 1,000 cycles of stretching and compressing at ambient temperature.

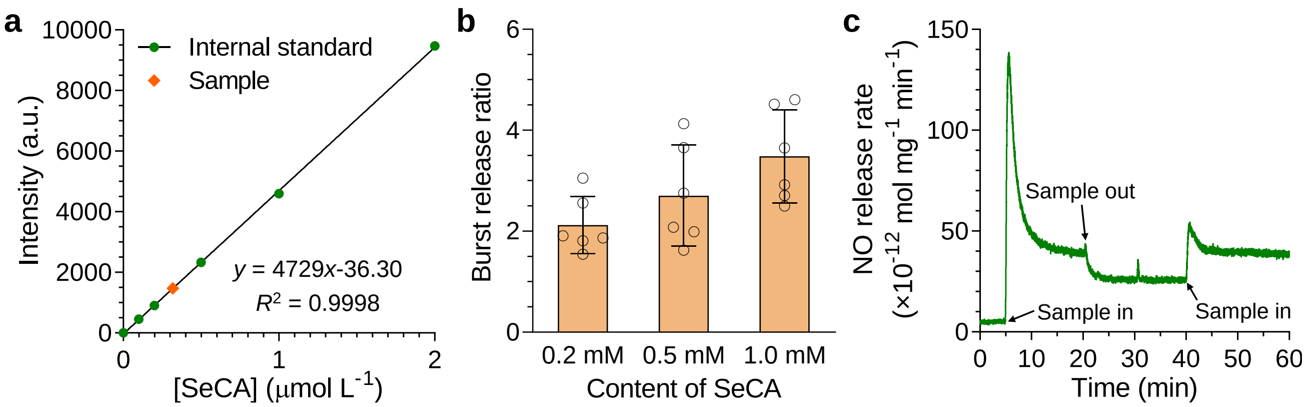

Supplementary Fig. 15 | Catalytic generation of nitric oxide (NO) in the hydrogel. a, Determination of selenocystamine (SeCA) conjugated to A-M(9.3) by inductively coupled plasma mass spectrometry (ICP-MS). The mass concentration of the work solution for SeCA-conjugated A-M(9.3) was 20 mg mL^{-1}. **b**, Quantification on the burst release ratios of NO catalyzed by the nitric oxide-eluting (NOE) hydrogels conjugated with different contents of SeCA (mean ± SD, n = 6 independent samples). **c**, Representative curve of NO release from S-nitrosoglutathione (GSNO, 10 μM in PBS at 37 °C) catalyzed by the NOE hydrogel conjugated with 1.0 mM SeCA. The release rate of NO declined upon the removal of the hydrogel from the solution, but then returned to the original value after re-submergence.

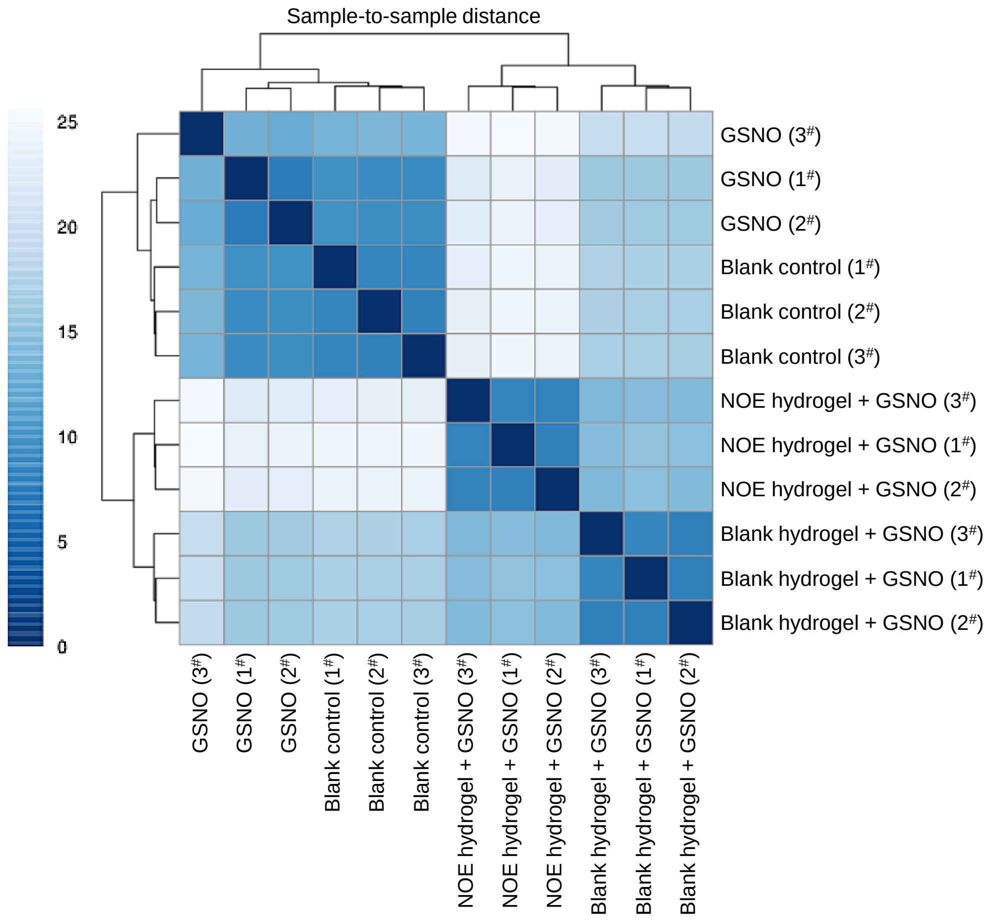

Supplementary Fig. 16 | Clustering assay showing the general variations in gene expression of human umbilical artery smooth muscle cells (HUASMCs) among different groups and replicates.

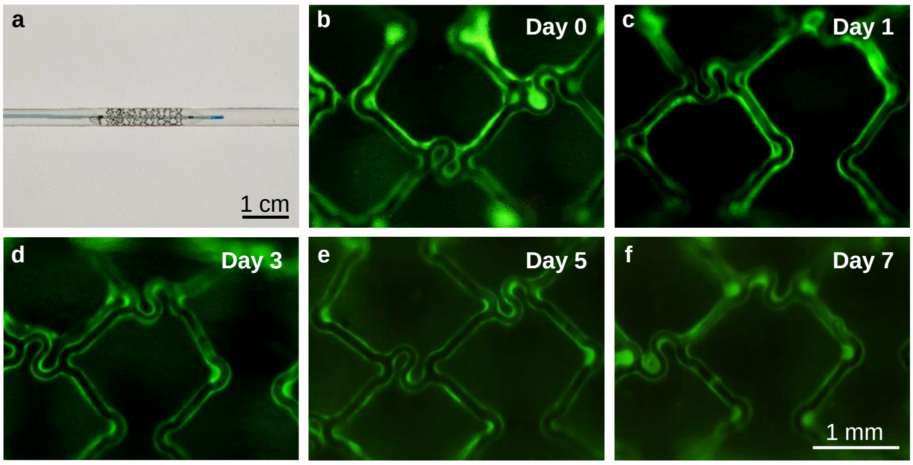

Supplementary Fig. 17 | Mechanical stability of the nitric oxide-eluting (NOE) hydrogel coating on a bare-metal stent (316L stainless steel) tested by mock angioplasty in a catheter. a, Photograph of the NOE hydrogel-coated stent implanted into a PVC catheter. **b-f**, Fluorescence images showing the preservation of the NOE hydrogel coating on the stent after being flushed by PBS at 37 °C for different durations. The NOE hydrogel was labeled with fluorescein isothiocyanate.

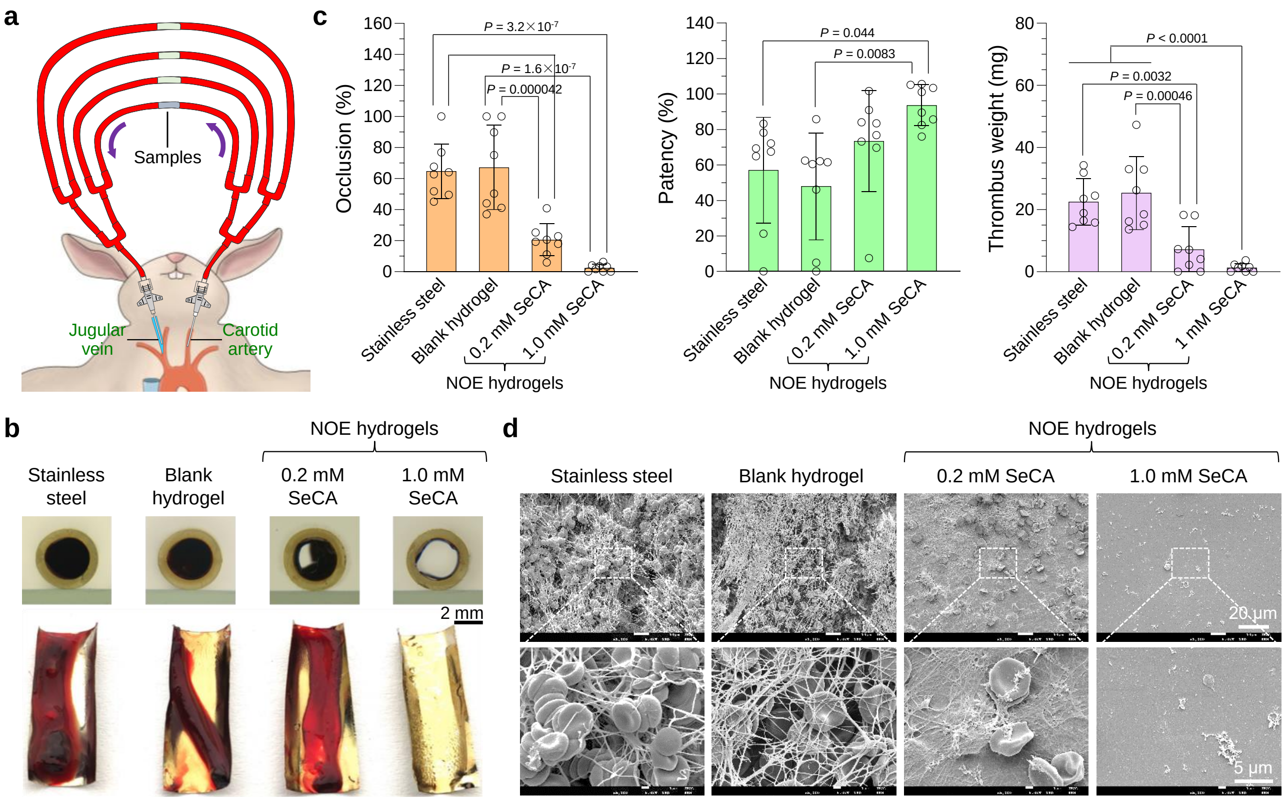

Supplementary Fig. 18 | *Ex vivo* thrombogenicity test of the nitric oxide-eluting (NOE) hydrogels. **a**, Schematic illustration for the arteriovenous extracorporeal circuit established on rabbits. **b**, Photographs of the thrombi formed on various substrates. (upper panel: cross-sections of the catheters containing the samples after the *ex vivo* thrombogenicity test; lower panel: thrombi on the expanded samples). **c**, Quantitative assessment on the thrombogenicities of various substrates (mean ± SD, n = 8 independent experiments). **d**, Scanning electron microscopy (SEM) images of thrombi on various substrates. One-way ANOVA with Tukey post-hoc test was performed to determine the difference among various groups.

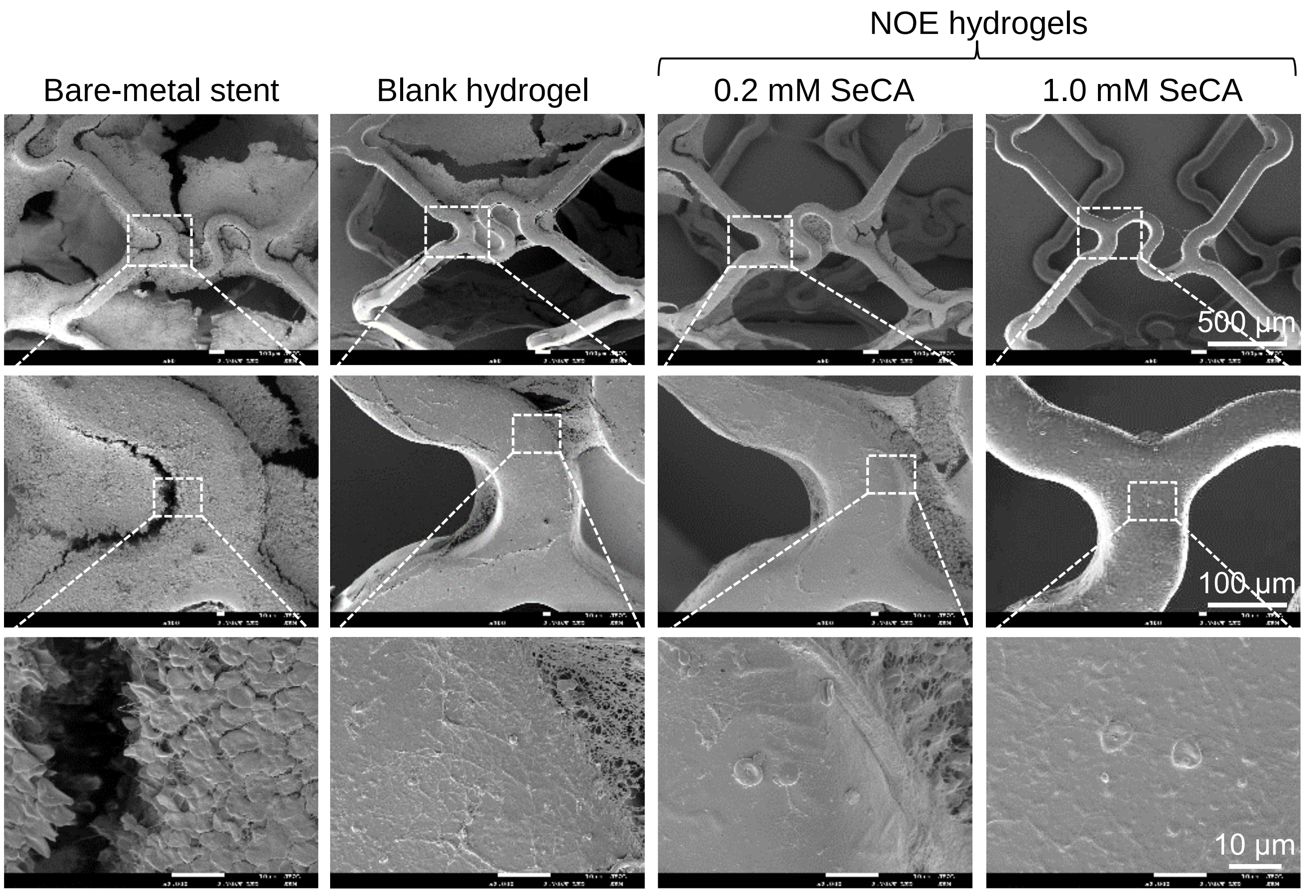

Supplementary Fig. 19 | Scanning electron microscopy (SEM) images showing the thrombus formation on the bare-metal stent (316L stainless steel) and the blank hydrogel- or NOE hydrogel-coated stents post the *ex vivo* thrombogenicity test. Three independent samples were observed with similar results for each group.

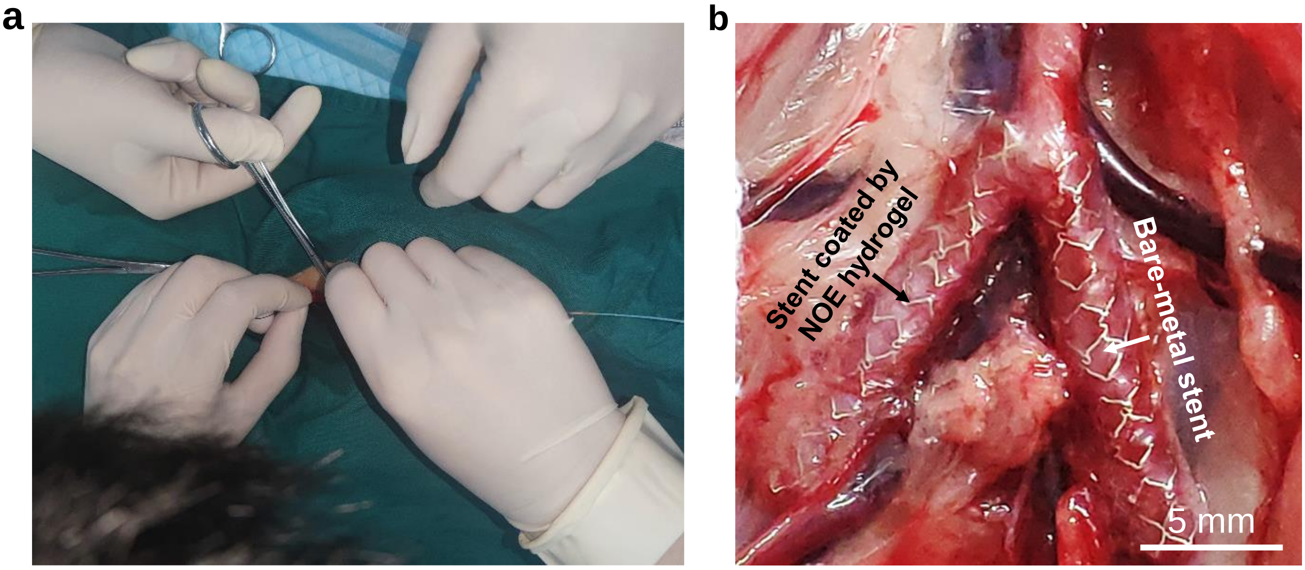

Supplementary Fig. 20 | Photos showing the process of stent implantation in rabbit iliac arteries (a) and the stented arteries (b).

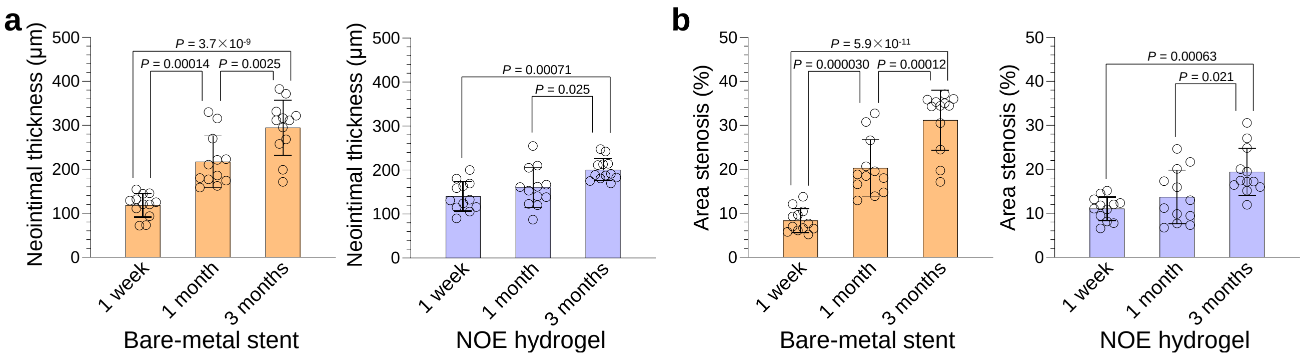

Supplementary Fig. 21 | Time-resolved quantitative analyses on neointimal hyperplasia of the stented rabbit iliac arteries (mean ± SD, n = 12 independent animals). One-way ANOVA with Tukey post-hoc test was performed to determine the difference among different times.

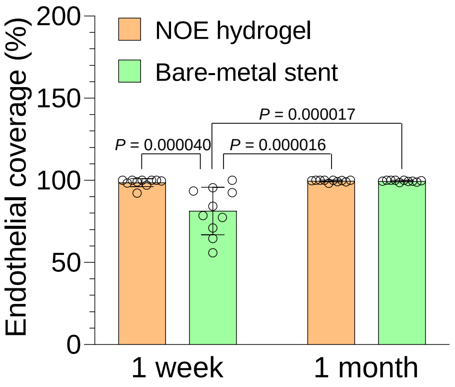

Supplementary Fig. 22 | Quantitative analysis on endothelial regeneration of the stented rabbit iliac arteries (mean ± SD, n = 10 independent animals). One-way ANOVA with Tukey post-hoc test was performed to determine the difference among different groups and times.

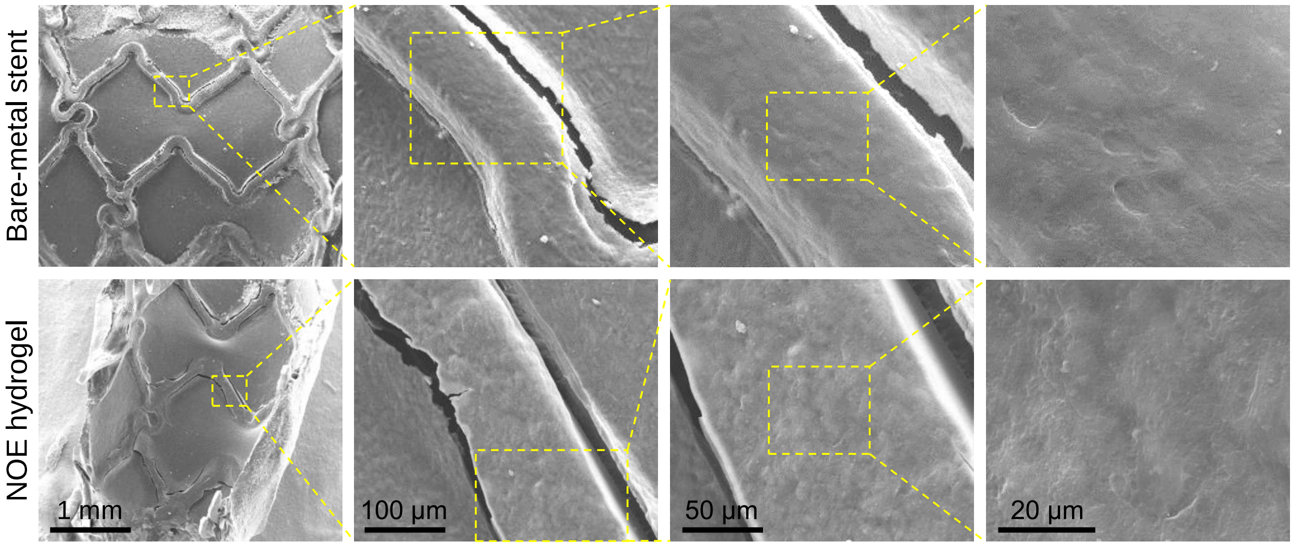

Supplementary Fig. 23 | Scanning electron microscopy (SEM) images showing the luminal faces of stented arteries at 1 week post stent deployment in a *New Zealand* rabbit. Three independent pairs of samples were observed with similar results.

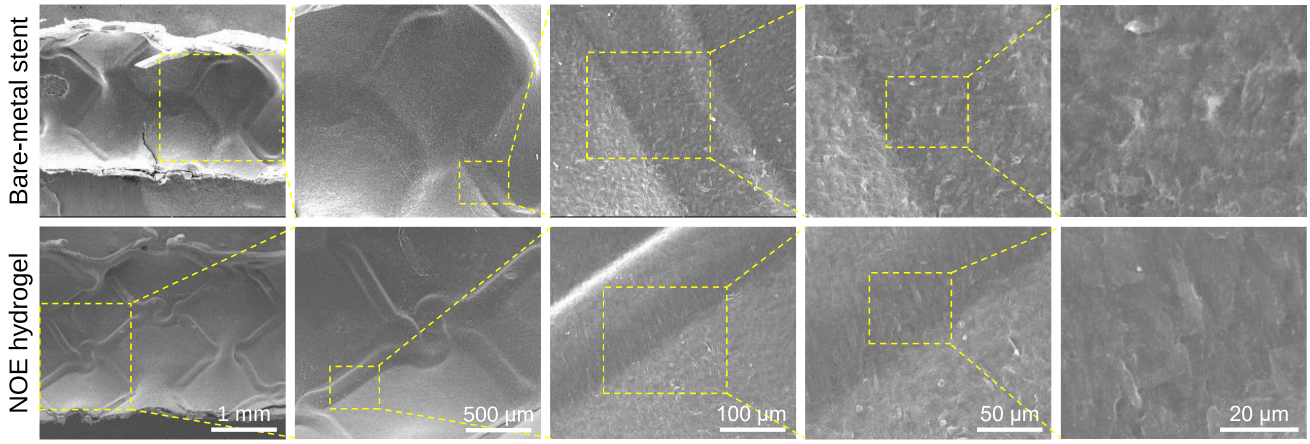

Supplementary Fig. 24 | Scanning electron microscopy (SEM) images showing the luminal faces of stented arteries at 1 month post stent deployment in a *New Zealand* rabbit. Three independent pairs of samples were observed with similar results.

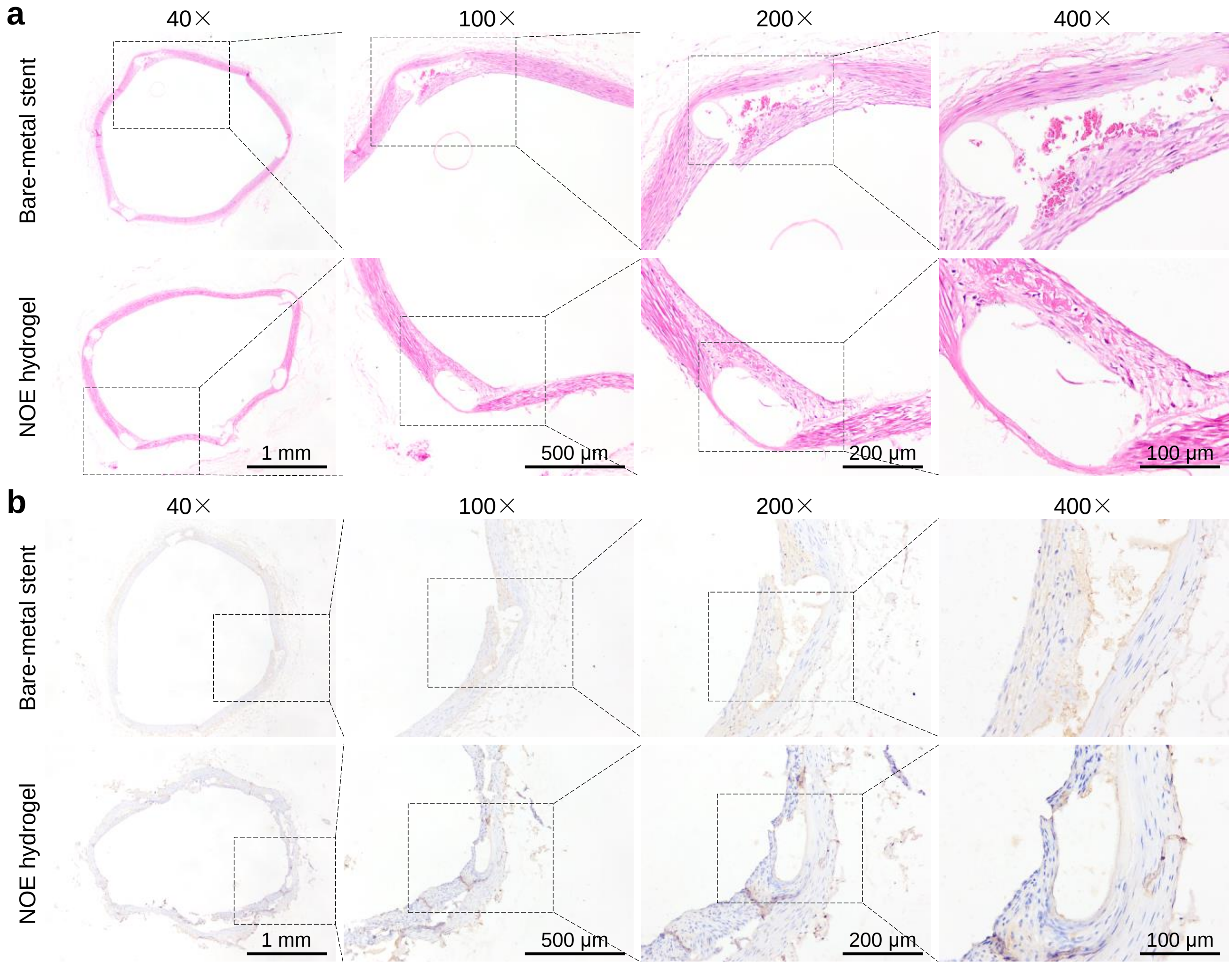

Supplementary Fig. 25 | Histological analyses on the stented rabbit iliac arteries at 1 week post stent deployment. a, Hematoxylin and eosin (H&E) staining on the cross-sections of the stented arteries. **b**, CD68 immunostaining on the cross-sections of the stented arteries. Three independent samples were observed with similar results for each group.

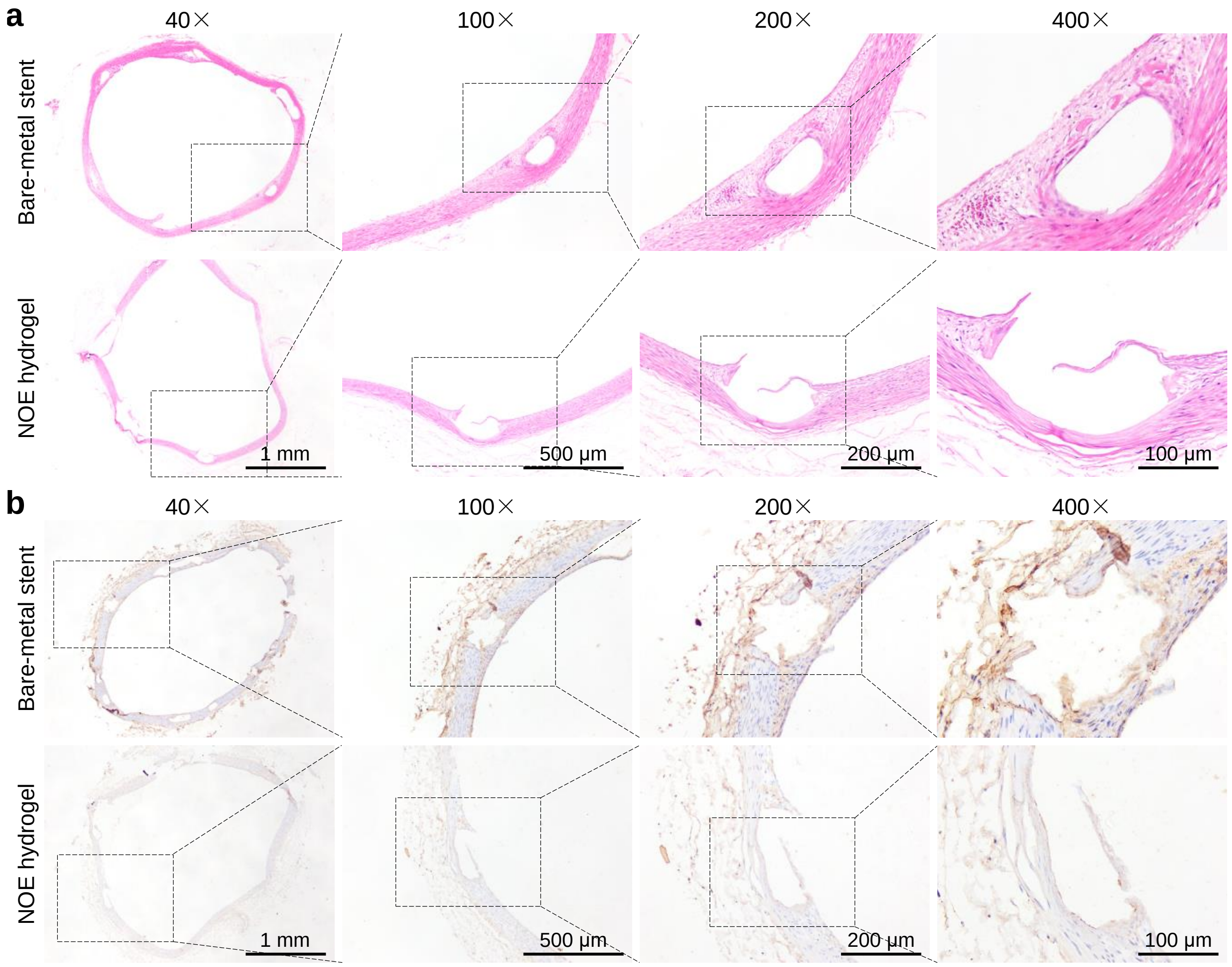

Supplementary Fig. 26 | Histological analyses on the stented rabbit iliac arteries at 1 month post stent deployment. a, Hematoxylin and eosin (H&E) staining on the cross-sections of the stented arteries. **b**, CD68 immunostaining on the cross-sections of the stented arteries. Three independent samples were observed with similar results for each group.

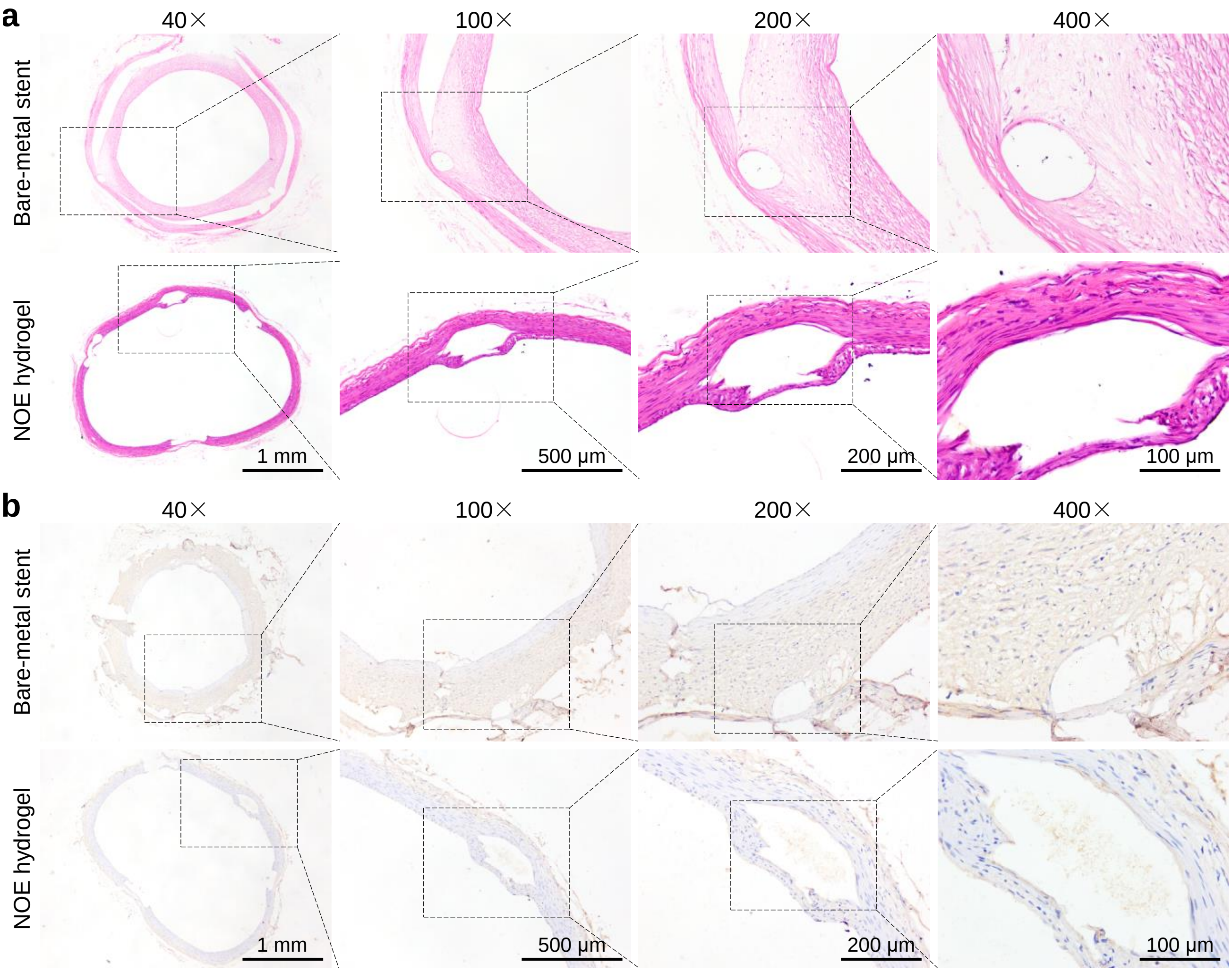

Supplementary Fig. 27 | Histological analyses on the stented rabbit iliac arteries at 3 months post stent deployment. a, Hematoxylin and eosin (H&E) staining on the cross-sections of the stented arteries. **b**, CD68 immunostaining on the cross-sections of the stented arteries. Three independent samples were observed with similar results for each group.

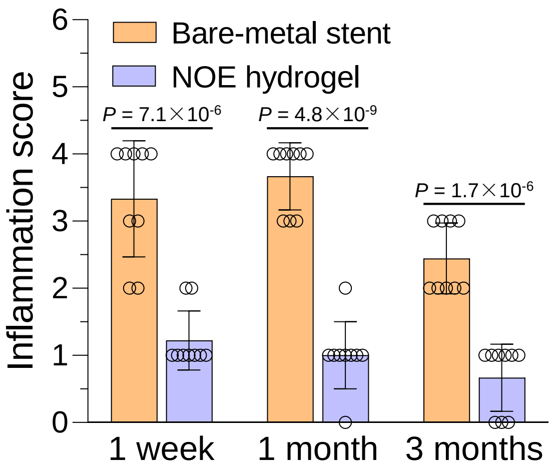

Supplementary Fig. 28 | Quantitative analysis on inflammation of the stented rabbit iliac arteries (mean ± SD, *n* = 9 struts examined over 3 independent animals). Two-tailed Student's *t*-test was performed to determine the difference between two groups.

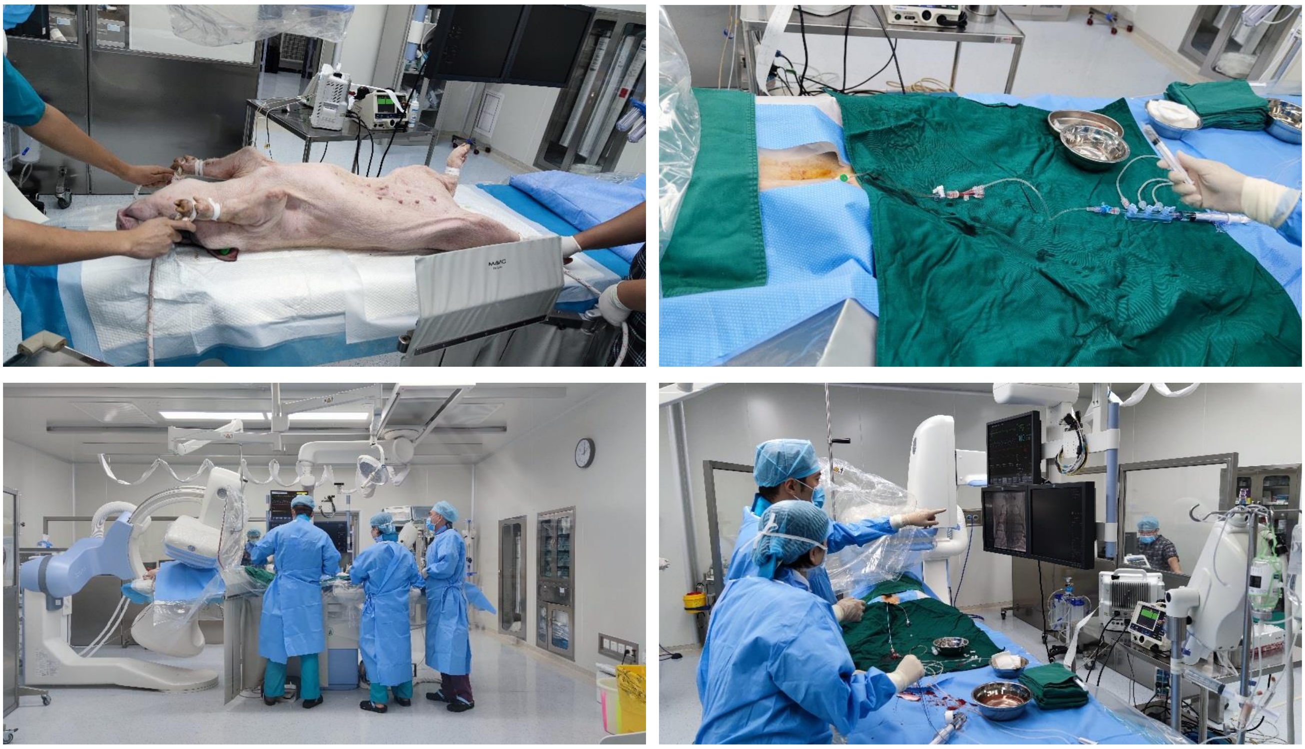

Supplementary Fig. 29 | Photos showing the process of stent implantation in the coronary arteries of a *Bama* miniature pig.

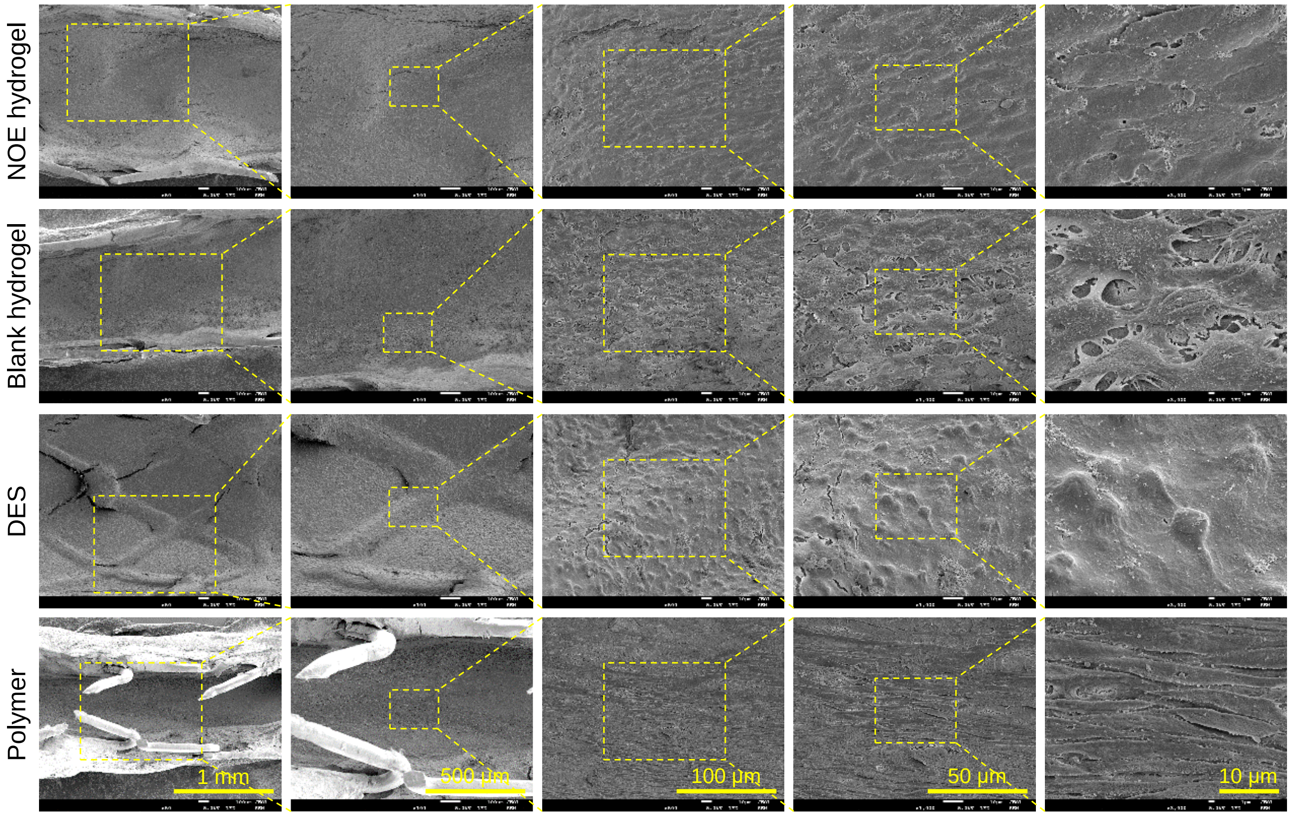

Supplementary Fig. 30 | Scanning electron microscopy (SEM) images showing the luminal faces of stented arteries at 2 weeks post stent deployment in a *Bama* miniature pig. Three independent samples were observed with similar results for each group.

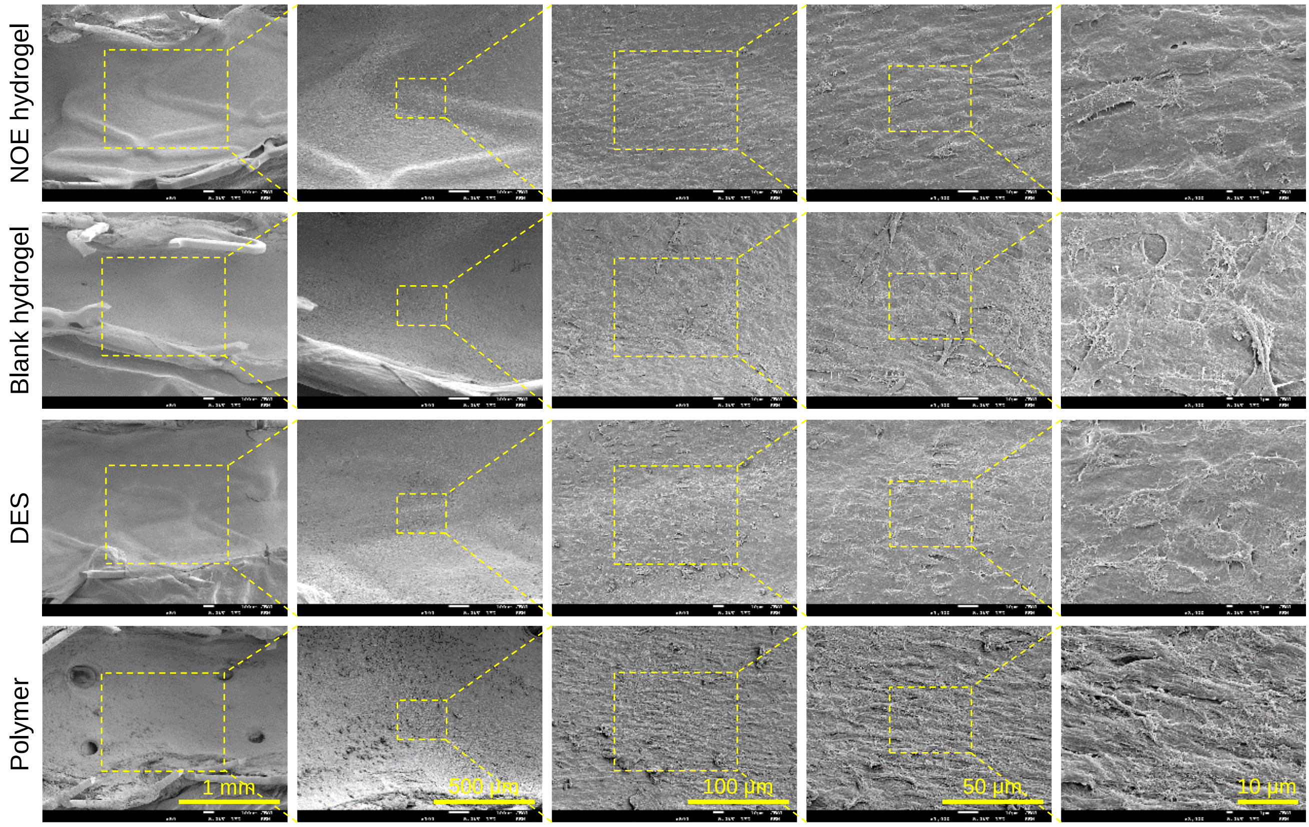

Supplementary Fig. 31 | Scanning electron microscopy (SEM) images showing the luminal faces of stented arteries at 3 months post stent deployment in a *Bama* miniature pig. Three independent samples were observed with similar results for each group.

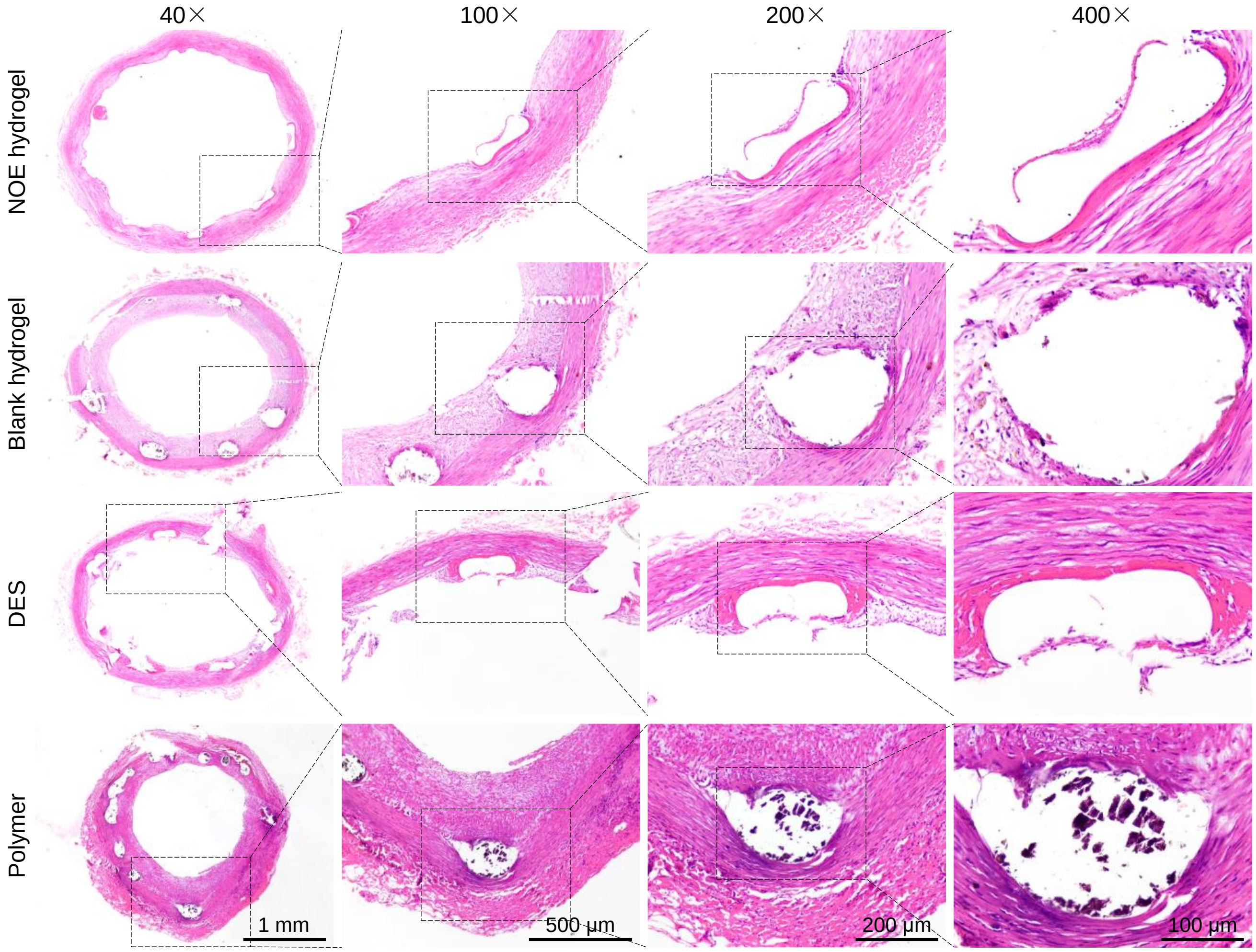

Supplementary Fig. 32 | Hematoxylin and eosin (H&E) staining on the cross-sections of the stented porcine coronary arteries at 2 weeks post stent deployment. Three independent samples were observed with similar results for each group.

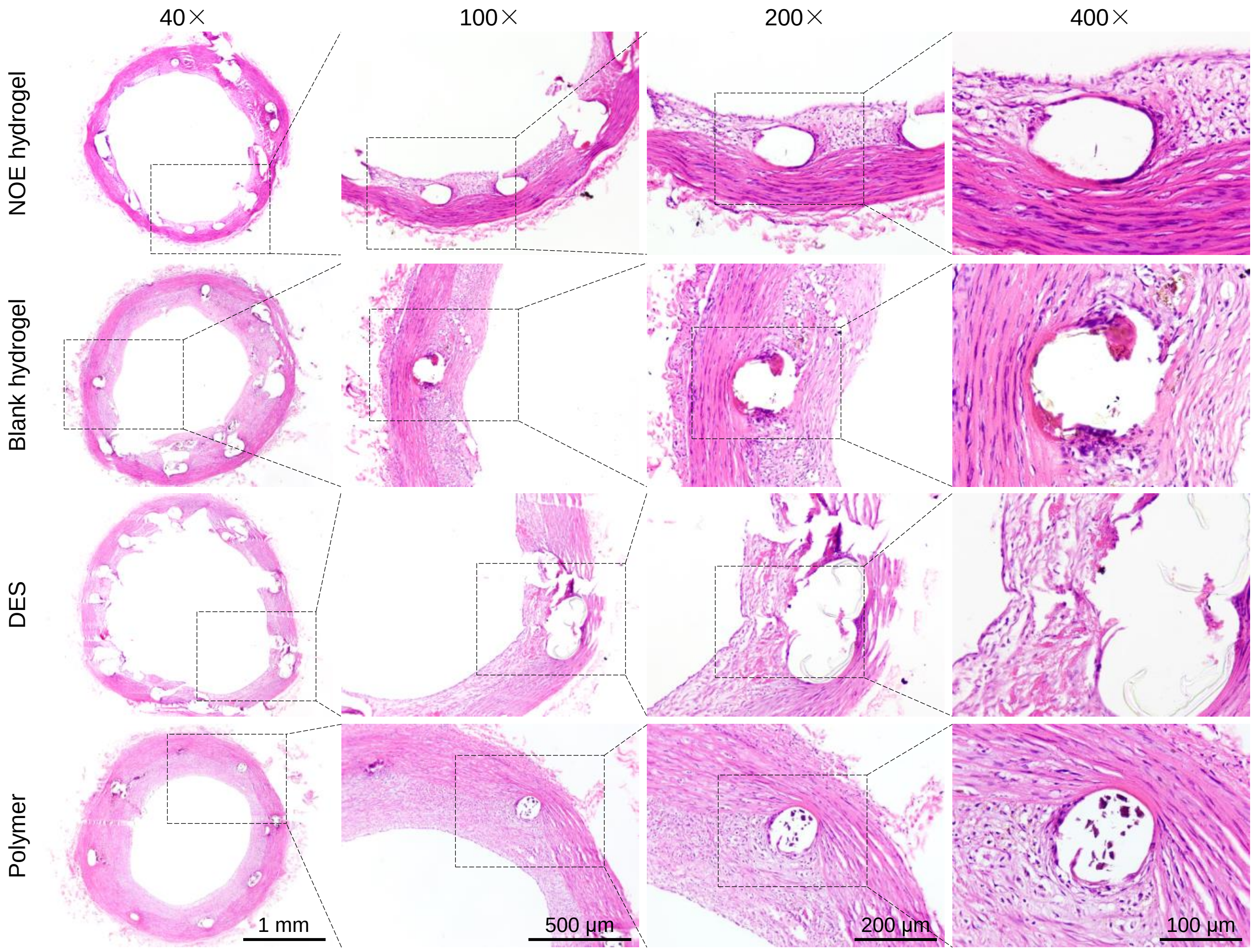

Supplementary Fig. 33 | Hematoxylin and eosin (H&E) staining on the cross-sections of the stented porcine coronary arteries at 3 months post stent deployment. Three independent samples were observed with similar results for each group.

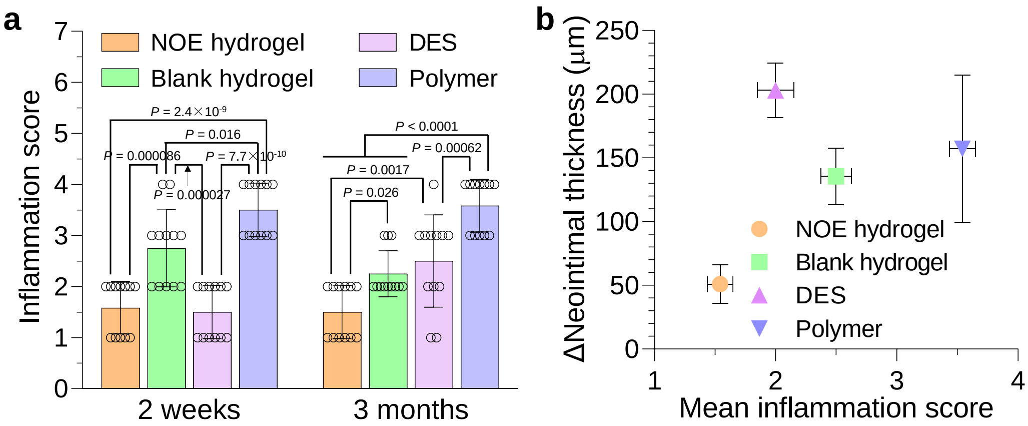

Supplementary Fig. 34 | Quantitative analysis on inflammation of the stented porcine coronary arteries (mean ± SD, *n* = 12 struts examined over 3 independent animals) (a) and its correlation with neointimal hyperplasia (mean ± SEM, *n* = 6 independent animals) (b). The increments in neointimal thickness between 2 weeks and 3 months post stent deployment were plotted versus the mean inflammation scores during that period of time. One-way ANOVA with Tukey post-hoc test was performed to determine the difference among different groups.

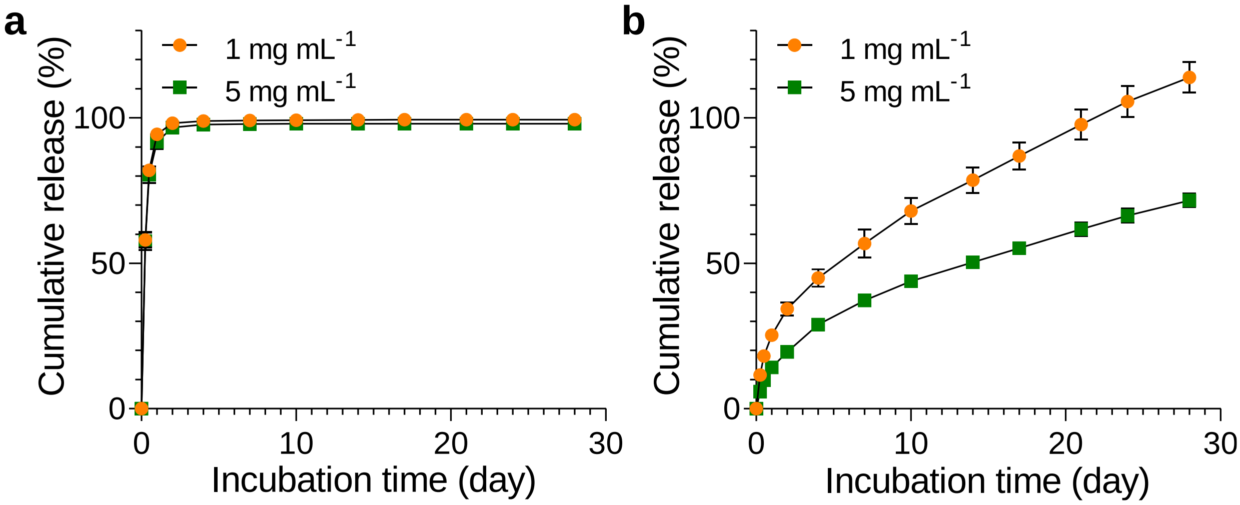

Supplementary Fig. 35 | Release profiles of Rhodamine B (a) and fluorescein isothiocyanate (FITC)-labeled bovine serum albumin (BSA) (b) from the nitric oxide-eluting (NOE) hydrogel incubated in PBS at 37 °C (mean ± SD, *n* = 8 independent samples).

Supplementary Table 1 | Events of stent thrombosis in rabbit iliac arteries

	1 week	1 month	3 months
Bare-metal stent	2	0	0
NOE hydrogel-coated stent	0	0	0

Supplementary Table 2 | Criteria for inflammation scoring

Attribute	Score	Description of assigned weight
No inflammation	0	No inflammatory cells
Minimal inflammation	1	0 < Inflammatory cells ≤ 5
Mild inflammation	2	5 < Inflammatory cells ≤ 10
Moderate inflammation	3	10 < Inflammatory cells ≤ 20
Severe inflammation	4	Inflammatory cells > 20

Supplementary Table 3 | Events of stent thrombosis in porcine coronary arteries

	2 weeks	3 months
NOE hydrogel-coated stent	0	0
Blank hydrogel-coated stent	1	0
DES	0	0
Polymer-coated stent	1	0

Supplementary Table 4 | Comparison of our stents with other stents

Name of stents	Stent designs			Thrombogenicity	Endothelialization			Area stenosis (%)		
	Stent base	Stent coating	Therapeutics		1 week	1 month	3 months	1 week	1 month	3 months
**I. Drug eluting stents (DESs)**										
**NOE hydrogel-coated stent (our data)**	**316L SS**	**Hydrogel**	**Nitric oxide**	**Low**	**Complete**	**Complete**	**Complete**	**11.1±2.7**	**13.7±6.1**	**19.4±5.4**
Akt1 siRNA-eluting stent[1]	CoCr	BRP	Akt1 siRNA	Low	NA	NA	NA	NA	9.4±1.6	NA
D65495-eluting stent[2]	CoCr	None	D65495	High	NA	Complete	Complete	NA	7.9±2.2	NA
Cerivastatin-eluting stent[3]	CoCr	Silica sol-gel	Cerivastatin	Low	NA	Complete	Complete	NA	15.0±6.0	NA
Gene-eluting stent[4]	SS	NBRP	7ND cDNA	Low	NA	Complete	Complete	NA	24.6±5.2	NA
Paclitaxel and NO-eluting stent[5]	SS	NBRP	Paclitaxel & Nitric oxide	Low	NA	NA	NA	NA	15.0±5.1	NA
Corolimus-eluting stent[6]	CoCr	Crosslinked omega-3 fatty acid	Corolimus	Low	NA	NA	NA	NA	NA	22.3±10.0
miR-145-eluting stent[7]	CoCr	BRP	miR-145	Low	NA	NA	NA	NA	15.2±5.2	NA
NO-eluting stent[8]	316L SS	EPC-targeting peptide	Nitric oxide	Low	Complete	Complete	Complete	NA	18.3±2.2	20.2±1.4
6-mercaptopurine-eluting stent[9]	CoCr	BRP	6-mercaptopurine	High	Complete	Complete	Complete	NA	14.9±1.0	NA
Sirolimus-eluting stent[10]	CoCr	NBRP	Sirolimus	Low	Incomplete	Incomplete	NA	NA	13.7±5.3	NA
Zotarolimus-eluting stent[10]	CoCr	NBRP	Zotarolimus	Low	Incomplete	Incomplete	NA	NA	13.7±6.1	NA
Everolimus-eluting stent[10]	CoCr	NBRP	Everolimus	Low	Incomplete	Incomplete	NA	NA	13.7±6.2	NA
NF-κB decoy-eluting stent[11]	SS	NBRP	NF-κB	High	NA	Complete	Complete	NA	41.5±6.2	NA
XIENCE V®[12]	CoCr	NBRP	Everolimus	Low	Incomplete	Incomplete	NA	NA	NA	NA
Atorvastatin-eluting stent[13]	CoCr	BRP	Atorvastatin	High	NA	NA	NA	NA	14.8±5.1	NA
Endeavor®[12,14]	CoCr	NBRP	Zotarolimus	Low	Incomplete	Incomplete	Incomplete	NA	14.3±4.1	15.4±2.4
Bevacizumab-eluting stent[15]	316L SS	Phosphorylcholine	Bevacizumab	Low	Incomplete	Incomplete	NA	NA	16.7±1.6	NA
Taxus Liberté®[12]	316L SS	NBRP	Paclitaxel	High	Incomplete	Incomplete	NA	NA	NA	NA
Synergy®[16]	PtCr	BRP	Everolimus	High	Incomplete	Incomplete	NA	NA	NA	NA
BioMatrix Flex®[16]	316L SS	BRP	Biolimus A9	High	Incomplete	Incomplete	NA	NA	NA	NA
Cypher®[12,14]	316L SS	NBRP	Sirolimus	High	Incomplete	Incomplete	Incomplete	NA	8.8±4.1	20.1±9.0
Taxus Element®[17]	PtCr	NBRP	Paclitaxel	High	Incomplete	Incomplete	NA	NA	21.6±5.8	NA
*c*RGD-eluting stent[18]	CoCr	None	*c*RGD peptide	High	Incomplete	Incomplete	NA	NA	23.4±7.0	NA
Taxus Express®[17]	316L SS	NBRP	Paclitaxel	High	Incomplete	Incomplete	NA	NA	25.2±7.1	NA
As_2O_3- eluting stent[19]	SS	BRP	Arsenic trioxide	High	Incomplete	Incomplete	NA	NA	26.1±6.1	NA
D24851-eluting stent[20]	SS	BRP	D24851	High	Incomplete	Incomplete	Incomplete	NA	20.2±5.2	NA
**II. Bare metal stents (BMSs)**										
**BMS (our control)**	316L SS	None	None	High	Incomplete	Complete	Complete	8.3±2.7	20.3±6.4	31.2±6.9
BMS[21]	Ti	None	None	Low	NA	NA	NA	NA	20.0±3.8	NA
Driver®[1]	CoCr	None	None	High	Incomplete	Complete	Complete	NA	19.5±9.7	10.0±2.8
R-Stent Evolution 2[22]	316L SS	None	None	High	NA	Complete	Complete	NA	23.3±11.3	NA
BMS[21]	SS	None	None	High	NA	NA	NA	NA	24.8±7.5	NA

Supplementary Table 4 | Comparison of our stents with other stents (Continued)

**II. Bare metal stents (BMSs) (Continued)**										
Coroflex Blue®[23]	CoCr	None	None	High	NA	NA	NA	NA	31.0±10.2	NA
CNUH[23]	CoCr	None	None	High	NA	NA	NA	NA	35.0±18.6	NA
ACS Multilink Duet®[24]	316L SS	None	None	High	NA	Complete	Complete	NA	25.9±1.2	31.9±4.4
MULTI-LINK Vision®[10]	CoCr	None	None	High	Incomplete	Complete	NA	NA	20.7±6.3	NA
Bx Velocity®[17]	316L SS	None	None	High	Incomplete	Incomplete	NA	NA	25.7±3.5	NA
**III. Fully bioresorbable stent**										
Absorb BVS®[16,25]	BRP	BRP	Everolimus	High	Incomplete	Incomplete	Incomplete	NA	NA	30.4±3.0
**IV. Other stents**										
REDV-immobilized stent[26]	316L SS	REDV-immobilized NBRP	None	Low	Complete	Complete	Complete	NA	41.0±1.9	NA
Anti-ApoA-I-immobilized stent[22]	316L SS	Anti-ApoA-I antibody	None	Low	NA	Complete	Complete	NA	23.3±13.8	NA
TiO_2 nanotube-coated stent[21]	Ti	TiO_2 nanotube	None	Low	NA	NA	NA	NA	17.2±1.1	NA
VEGF-immobilized stent[27]	316L SS	VEGF-immobilized BRP	None	High	NA	Complete	Complete	NA	42.6±5.0	NA
Plasma polymer-coated stent[28]	316L SS	Plasma polymer	None	Low	Incomplete	NA	NA	12.1±1.2	NA	NA
TENAX®[29]	316L SS	Silicon carbide	None	High	NA	NA	NA	NA	15.2±3.1	NA
BX Isostent®[30]	SS	^{32}P coating	β particle	High	Incomplete	Incomplete	Incomplete	NA	NA	11.5±3.0
Heparin-immobilized stent[31]	CoCr	Heparin-immobilized BRP	None	Low	Incomplete	Incomplete	NA	NA	39.0±28.7	NA

SS: Stainless steel; CoCr: Cobalt chromium alloy; PtCr: Platinum chromium alloy; Ti: Titanium; BRP: Bioresorbable polymer; NBRP: Non-bioresorbalbe polymer; NA: Not available

(yellow) Property inferior to that of our NOE hydrogel-coated stent

(green) Property comparable to that of our NOE hydrogel-coated stent

(cyan) Property superior to that of our NOE hydrogel-coated stent

Thrombogenicities of the NOE hydrogels

Blood coagulation is a mechanism to stop bleeding and prevent overexposure to infectious agents in the environment when injury occurs. However, it is also a barrier for angioplasty because the unavoidable damage to vessel wall will induce the release of tissue factors that can activate thrombin and platelets[32], thereby triggering clotting cascade on the implant. NO is capable of activating guanylate cyclase, which catalyzes the production of cyclic guanosine monophosphate (cGMP) that can inhibit the activation and aggregation of platelets[33]. Due to the capacity to catalyze the degradation of RSNO into NO, our NOE hydrogels were supposed to possess anti-coagulant potency upon the supplementation of NO donors in blood. To assess their thrombogenicities, we established an arteriovenous extracorporeal circuit model on rabbits (Supplementary Fig. 18a). The circuit consisted of four catheters that were connected to a carotid artery and a jugular vein of a rabbit through two connecting hoses. Each of the four catheters contained a specimen and was constantly flushed by blood flow during the test.

After the *ex vivo* test, catheters containing a bare stainless steel foil were severely occluded by thrombi (Supplementary Fig. 18b). For those foils with a hydrogel coating, thrombus formation varied dramatically. The thrombogenicity of the blank hydrogel seemed to be on par with that of bare stainless steel. In fact, quantitative assessment (Supplementary Fig. 18c) indicated no significant difference between bare stainless steel and the blank hydrogel in terms of the occlusion and patency of the host catheter, as well as the thrombus weight on the foil. However, with the introduction of SeCA, the NOE hydrogels became anti-coagulant. For the NOE hydrogel conjugated with 0.2 mM SeCA, the occlusion of the host catheter decreased from 67.2% to 20.3% ($P < 0.0001$) with the thrombus weight reduced from 25.4 mg to 6.8 mg ($P < 0.0001$), whereas the patency of it increased from 47.9% to 73.5%. With

the content of conjugated SeCA increasing to 1.0 mM, the NOE hydrogel became even non-thrombogenic as almost no thrombus could be seen on any specimen of it with naked eyes (Supplementary Fig. 18b). Consequently, the host catheters presented low occlusion (2.4%) and high patency (93.9%) after the *ex vivo* test, approaching the values of empty ones. Scanning electron microscope (SEM) displayed the magnified details of the thrombi formed on various substrates (Supplementary Fig. 18d). The thrombi on the bare stainless steel foil and blank hydrogel were mainly thick aggregations of erythrocytes interwoven with dense filaments. These filaments were believed be the assemblies of fibrin originating from the hydrolysis of fibrinogen catalyzed by thrombin, which played an important role in the process of blood coagulation[34]. In stark contrast, the thrombus formed on the NOE hydrogel conjugated with 0.2 mM SeCA was only a thin layer of filaments dispersed with sparse erythrocytes and tiny floccules. These floccules might be the precipitates of some plasma proteins, but we were unable to determine their composition. Excitingly, when the content of conjugated SeCA increased to 1.0 mM, only sporadic blood cells and floccules were found on the NOE hydrogel.

To demonstrate that the NOE hydrogels also function on the vascular stents, we performed the *ex vivo* test to compare the bare-metal stent and hydrogel-coated stents directly. As expected, similar trends were observed when the stents were tested (Supplementary Fig. 19). Taken together, these results confirmed the NO generation catalyzed by the NOE hydrogel could effectively retard the process of blood coagulation or even completely inhibit it depending on the content of conjugated SeCA.

References

1. Che, H.-L. et al. Therapeutic effect of Akt1 siRNA nanoparticle eluting coronary stent on suppression of post-angioplasty restenosis. *J. Biomed. Nanotechnol.* **12**, 1211-1222 (2016).
2. Jandt, E. et al. Stent-based release of a selective PDGF-receptor blocker from the bis-indolylmethanon class inhibits restenosis in the rabbit animal model. *Vasc. Pharmacol.* **52**, 55-62 (2010).
3. Pendyala, L.K. et al. Polymer-free cerivastatin-eluting stent shows superior neointimal inhibition with preserved vasomotor function compared to polymer-based paclitaxel-eluting stent in rabbit iliac arteries. *Eurointervention* **6**, 126-133 (2010).
4. Egashira, K. et al. Local delivery of anti-monocyte chemoattractant protein-1 by gene-eluting stents attenuates in-stent stenosis in rabbits and monkeys. *Arterioscler., Thromb., Vasc. Biol.* **27**, 2563-2568 (2007).
5. Lin, C.-E. et al. Combination of paclitaxel and nitric oxide as a novel treatment for the reduction of restenosis. *J. Med. Chem.* **47**, 2276-2282 (2004).
6. Artzi, N. et al. Sustained efficacy and arterial drug retention by a fast drug eluting cross-linked fatty acid coronary stent coating. *Ann. Biomed. Eng.* **44**, 276-286 (2016).
7. Che, H.-L. et al. Novel fabrication of microRNA nanoparticle-coated coronary stent for prevention of post-angioplasty restenosis. *Korean Circ. J.* **46**, 23-32 (2016).
8. Yang, Z. et al. Bioclickable and mussel adhesive peptide mimics for engineering vascular stent surfaces. *Proc. Natl. Acad. Sci. USA* **117**, 16127-16137 (2020).
9. Ruiter, M.S. et al. Stents eluting 6-mercaptopurine reduce neointima formation and inflammation while enhancing strut coverage in rabbits. *PLoS One* **10**, e0138459 (2015).
10. Steigerwald, K. et al. Vascular healing in drug-eluting stents: differential drug-associated response of limus-eluting stents in a preclinical model of stent implantation. *Eurointervention* **8**, 752-759 (2012).
11. Ohtani, K. et al. Stent-based local delivery of nuclear factor-κB decoy attenuates in-stent restenosis in hypercholesterolemic rabbits. *Circulation* **114**, 2773-2779 (2006).
12. Joner, M. et al. Endothelial cell recovery between comparator polymer-based drug-eluting stents. *J. Am. Col.l Cardiol.* **52**, 333-342 (2008).
13. Lim, K.S. et al. Effect of atorvastatin-eluting stents in a rabbit iliac artery restenosis model. *Chonnam Med. J.* **49**, 118-124 (2013).
14. Nakazawa, G., Finn, A.V., John, M.C., Kolodgie, F.D. & Virmani, R. The significance of preclinical evaluation of sirolimus-, paclitaxel-, and zotarolimus-eluting stents. *Am. J. Cardiol.* **100**, S36-S44 (2007).
15. Stefanadis, C. et al. Inhibition of plaque neovascularization and intimal hyperplasia by specific targeting vascular endothelial growth factor with bevacizumab-eluting stent: an experimental study. *Atherosclerosis* **195**, 269-276 (2007).
16. Koppara, T. et al. Thrombogenicity and early vascular healing response in metallic biodegradable polymer-based and fully bioabsorbable drug-eluting stents. *Circ.: Cardiovasc. Interventions* **8**, e002427 (2015).
17. Finn, A.V. et al. Differential response of delayed healing and persistent inflammation at sites of overlapping sirolimus- or paclitaxel-eluting stents. *Circulation* **112**, 270-278 (2005).
18. Rechenmacher, F. et al. The integrin ligand *c*(RGDf(*N*Me)Nal) reduces neointimal hyperplasia in a polymer-free drug-eluting stent system. *ChemMedChem* **9**, 1413-1418 (2014).
19. Yang, W. et al. Arsenic trioxide eluting stent reduces neointima formation in a rabbit iliac artery injury model. *Cardiovasc. Res.* **72**, 483-493 (2006).
20. Lysitsas, D.N. et al. Antirestenotic effects of a novel polymer-coated d-24851 eluting stent. experimental data in a rabbit iliac artery model. *Cardiovasc. Intervent. Radiol.* **30**, 1192-1200 (2007).
21. Nuhn, H., Blanco, C.E. & Desai, T.A. Nanoengineered stent surface to reduce in-stent restenosis *in vivo*. *ACS Appl.*

Mater. Interfaces **9**, 19677-19686 (2017).

22. Strang, A.C. et al. Effect of anti-ApoA-I antibody-coating of stents on neointima formation in a rabbit balloon-injury model. *PLoS One* **10**, e0122836 (2015).
23. Bae, I.H. et al. Mechanical behavior and *in vivo* properties of newly designed bare metal stent for enhanced flexibility. *J. Ind. Eng. Chem.* **21**, 1295-1300 (2015).
24. Kolodgie, F.D. et al. Sustained reduction of in-stent neointimal growth with the use of a novel systemic nanoparticle paclitaxel. *Circulation* **106**, 1195-1198 (2002).
25. Vorpahl, M. et al. Vascular healing and integration of a fully bioresorbable everolimus-eluting scaffold in a rabbit iliac arterial model. *Eurointervention* **10**, 833-841 (2014).
26. Wei, Y. et al. Surface engineering of cardiovascular stent with endothelial cell selectivity for *in vivo* re-endothelialisation. *Biomaterials* **34**, 2588-2599 (2013).
27. Wang, J.-L. et al. Electropolymerization of dopamine for surface modification of complex-shaped cardiovascular stents. *Biomaterials* **35**, 7679-7689 (2014).
28. Waterhouse, A. et al. *In vivo* biocompatibility of a plasma-activated, coronary stent coating. *Biomaterials* **33**, 7984-7992 (2012).
29. Robert, R. et al. Experimental assessment of new stent technologies: validation of a comparative paired rabbit iliac artery study model. *J. Biomed. Mater. Res., Part B* **70B**, 303-310 (2004).
30. Farb, A., Tang, A.L., Shroff, S., Sweet, W. & Virmani, R. Neointimal responses 3 months after ^{32}P β-emitting stent placement. *Int. J. Radiat. Oncol., Biol., Phys.* **48**, 889-898 (2000).
31. Bae, I.-H., Park, I.-K., Park, D.S., Lee, H. & Jeong, M.H. Thromboresistant and endothelialization effects of dopamine-mediated heparin coating on a stent material surface. *J. Mater. Sci.: Mater. Med.* **23**, 1259-1269 (2012).
32. Mackman, N., Tilley, R.E. & Key, N.S. Role of the extrinsic pathway of blood coagulation in hemostasis and thrombosis. *Arterioscler., Thromb., Vasc. Biol.* **27**, 1687-1693 (2007).
33. Bellamy, T.C., Wood, J. & Garthwaite, J. On the activation of soluble guanylyl cyclase by nitric oxide. *Proc. Natl. Acad. Sci. USA* **99**, 507-510 (2002).
34. Kattula, S., Byrnes, J.R. & Wolberg, A.S. Fibrinogen and fibrin in hemostasis and thrombosis. *Arterioscler., Thromb., Vasc. Biol.* **37**, e13-e21 (2017).
