# Supplementary material for: A tough nitric oxide-eluting hydrogel coating suppresses neointimal hyperplasia on vascular stent
Source: Nat Commun. 2021 Dec 6;12:7079. doi: 10.1038/s41467-021-27368-4 (PMC8648853; doi:10.1038/s41467-021-27368-4)
Supplement: Supplementary file 3 — Description of Additional Supplementary Files [file 41467_2021_27368_MOESM3_ESM.docx]

**Description of Additional Supplementary Files:**

**Supplementary Video 1:** **Tensile testing of an A-M(9.3)/G_4/6 hydrogel.** The test was conducted on the hydrogel by stretching it at the rate of 0.1 mm s^-1^ until fracture. Time is shown in h:min:sec.

**Supplementary Video 2:** **Movie showing the 3D view of neointimal formation around a strut of a nitric oxide-eluting (NOE) hydrogel-coated stent at 1 week post implantation in the right iliac artery of a rabbit.** The movie was generated by Z-stacking of confocal laser scanning microscopy (CLSM) images. The cell nuclei, F-actin, and CD31 were stained with DAPI, phalloidin-TRITC, and mouse anti-rabbit CD31 antibody followed by Alexa Fluor^®^ 488-conjugated goat anti-mouse IgG secondary antibody, respectively. (blue: cell nucleus, green: CD31, red: F-actin).

**Supplementary Video 3:** **Movie showing the 3D view of neointimal formation around a strut of a 316L stainless steel bare-metal stent at 1 week post implantation in the left iliac artery of the same rabbit.** The movie was generated by Z-stacking of CLSM images. The cell nuclei, F-actin, and CD31 were stained with DAPI, phalloidin-TRITC, and mouse anti-rabbit CD31 antibody followed by Alexa Fluor^®^ 488-conjugated goat anti-mouse IgG secondary antibody, respectively. (blue: cell nucleus, green: CD31, red: F-actin).

**Supplementary Video 4:** **Digital subtraction angiography (DSA) showing blood flow in left anterior descending (LAD) artery and left circumflex (LCX) artery of a *Bama* miniature pig at 3 months post stent deployment.**

**Supplementary Video 5:** **DSA showing blood flow in right coronary artery (RCA) of the same *Bama* miniature pig at 3 months post stent deployment.**
